# Functional annotation of rare gene aberration drivers of pancreatic cancer

**DOI:** 10.1038/ncomms10500

**Published:** 2016-01-25

**Authors:** Yiu Huen Tsang, Turgut Dogruluk, Philip M. Tedeschi, Joanna Wardwell-Ozgo, Hengyu Lu, Maribel Espitia, Nikitha Nair, Rosalba Minelli, Zechen Chong, Fengju Chen, Qing Edward Chang, Jennifer B. Dennison, Armel Dogruluk, Min Li, Haoqiang Ying, Joseph R. Bertino, Marie-Claude Gingras, Michael Ittmann, John Kerrigan, Ken Chen, Chad J. Creighton, Karina Eterovic, Gordon B. Mills, Kenneth L. Scott

**Affiliations:** 1 Department of Molecular and Human Genetics, Baylor College of Medicine, One Baylor Plaza, Houston, Texas 77030, USA; 2 Rutgers Cancer Institute of New Jersey, 195 Little Albany Street, New Brunswick, New Jersey 08903, USA; 3 Department of Systems Biology, University of Texas M.D. Anderson Cancer Center, 1515 Holcombe Boulevard, Houston, Texas 77030, USA; 4 Department of Bioinformatics and Computational Biology, University of Texas M.D. Anderson Cancer Center, 1515 Holcombe Boulevard, Houston, Texas 77030, USA; 5 Dan L. Duncan Cancer Center, Baylor College of Medicine, One Baylor Plaza, Houston, Texas 77030, USA; 6 Department of Genomics Medicine, University of Texas M.D. Anderson Cancer Center, 1515 Holcombe Boulevard, Houston, Texas 77030, USA; 7 Department of Medicine, The University of Oklahoma Health Sciences Center, Oklahoma City, Oklahoma 73104, USA; 8 Human Genome Sequencing Center, Baylor College of Medicine, One Baylor Plaza, Houston, Texas 77030, USA; 9 Department of Pathology and Immunology, Baylor College of Medicine, One Baylor Plaza, Houston, Texas 77030, USA; 10 Department of Medicine, Baylor College of Medicine, One Baylor Plaza, Houston, Texas 77030, USA

## Abstract

As we enter the era of precision medicine, characterization of cancer genomes will directly influence therapeutic decisions in the clinic. Here we describe a platform enabling functionalization of rare gene mutations through their high-throughput construction, molecular barcoding and delivery to cancer models for *in vivo* tumour driver screens. We apply these technologies to identify oncogenic drivers of pancreatic ductal adenocarcinoma (PDAC). This approach reveals oncogenic activity for rare gene aberrations in genes including *NAD Kinase* (*NADK*), which regulates NADP(H) homeostasis and cellular redox state. We further validate mutant *NADK*, whose expression provides gain-of-function enzymatic activity leading to a reduction in cellular reactive oxygen species and tumorigenesis, and show that depletion of wild-type *NADK* in PDAC cell lines attenuates cancer cell growth *in vitro* and *in vivo*. These data indicate that annotating rare aberrations can reveal important cancer signalling pathways representing additional therapeutic targets.

## Abstract (web summary)

Next generation sequencing allows the identification of oncogenic driver genes in pancreatic cancer. Here, in an effort to identify additional causal genes, the authors develop a high throughput *in vivo* screen and identify genes that whilst infrequently mutated in pancreatic cancer contribute to tumour formation.

## Graphical abstract

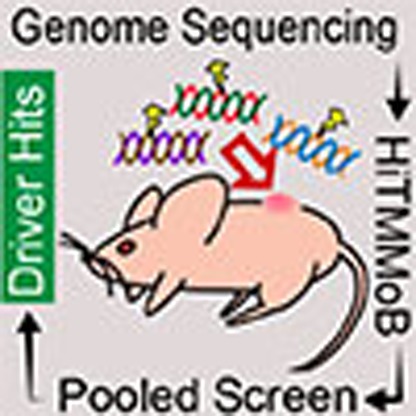

Large-scale efforts such as The Cancer Genome Atlas and International Cancer Genome Consortium (ICGC) are generating a compendium of genomic aberrations across major cancer types. Such studies are revealing the complexity of cancer genomes, which contain pathogenic driver aberrations and many more biologically neutral passenger events. While most cancers acquire one or more well-studied, high-frequency drivers, much less is known about which and how the abundant low-frequency variants contribute to tumour progression. Experimental assessment of low-frequency aberrations, in both under-characterized genes or among the long ‘tail' mutations observed in known cancer genes, is difficult, given their large number and the fact that they may either directly drive tumour progression or indirectly influence tumour behaviour through modifying activities of other driver aberrations. Moreover, distinguishing driver events from passengers is further complicated by the fact that driver activity is shaped by the biological context of a given cancer, including its tissue type, microenvironment and other host determinants. Identifying rare driver aberrations therefore requires robust pipelines that allow context-specific functional prioritization of the thousands of potential targets emerging from Next Generation Sequencing (NGS) data.

Here we describe a gain-of-function (GOF) screening platform enabled by a High-Throughput Mutagenesis and Molecular Barcoding (HiTMMoB) method allowing cost-effective, rapid modelling and expression of molecularly barcoded mutant gene variants into open reading frame (ORF) expression clones for pooled functional screens. HiTMMoB permits annotation of mutation-activated oncogenes, whose identification is particularly desirable, given the efficacy of antibody and small molecule inhibitor therapies that may be tailored towards such proteins. Our approach complements gene depletion and knockout strategies by RNA interference (RNAi) and CRISPR/Cas9-based platforms, respectively, which have been tremendously successful for identifying and validating tumour suppressor genes and other genetic dependencies^1^,^2^. HiTMMoB builds on previous over-expression screen-based studies designed to identify wild-type ORFs whose expression promotes tumorigenesis^3^,^4^, drug resistance^5^ and other cancer cell phenotypes such as anchorage-independent growth^6^,^7^,^8^ among others. Such screens are made possible through efforts by the ORFeome collaboration (http://www.orfeomecollaboration.org/; refs 9, 10, 11) and others^12^ who have systematically cloned and sequenced-validated a large majority of expressed human transcripts.

In this study we use HiTMMoB to identify low-frequency mutational events in pancreatic ductal adenocarcinoma (PDAC) that serve as drivers or effectors for recurrent drivers such as *KRAS*, a proto-oncogene activated in >90% of PDAC cases^13^. We employed HiTMMoB to build PDAC aberration libraries, whose compositions were based on individual PDAC patient genomes and computational selection, to identify rare oncogenic events that, when expressed in a human pancreatic cell model and implanted into mice, promotes *in vivo* tumour formation. This approach revealed potent activity for aberrations in genes including *NAD Kinase* (*NADK*) that regulates NADP(H) homeostasis and cellular redox state. We show that mutant *NADK* provides GOF enzymatic activity, leading to a reduction in cellular reactive oxygen species (ROS) and tumorigenesis. In addition, we show that depletion of wild-type *NADK* in PDAC cell lines attenuated cancer cell growth *in vitro* and *in vivo*. We conclude that barcode (BC)-assisted, GOF screening has the potential to have an impact on the field of cancer target discovery as a whole, particularly with respect to identifying low-frequency somatic events by complementing structural characterizations of cancer genomes with functional annotation of gene aberrations identified from tumour sequencing programmes.

## Results

### HiTMMoB method

**Figure 1:**
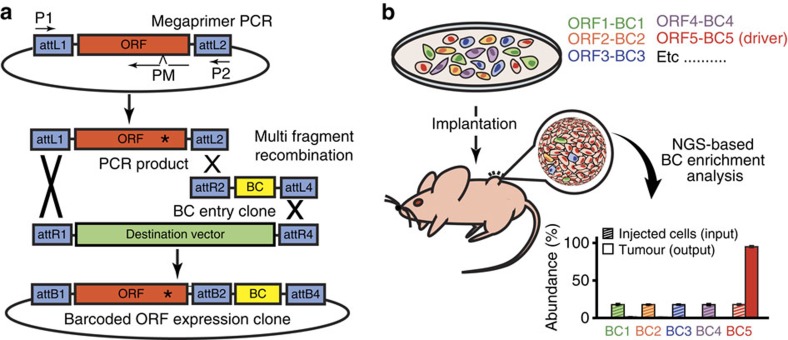
Functional annotation of cancer gene aberration HiTMMoB. Illustration of (**a**) HiTMMoB-mediated mutagenesis and molecular barcoding, and (**b**) barcode enrichment analysis. See text for details. BC, barcode; P1/P2/PM, PCR primers; attL/R/B, recombination sequences.

Successful translation of NGS data requires knowledge on which DNA aberrations represent actionable events, either for development or re-positioning of approved agents to target their activated pathways. To circumvent technical bottlenecks related to scaled construction of aberration expression clones, we developed a cost-effective HiTMMoB method (Fig. 1a) that employs: (1) two common PCR primers (P1/P2) flanking the ORF gene target and a third primer containing the desired mutation (PM), (2) a three-stage PCR reaction, whose design incorporates differential P1/P2 and PM melting temperatures^14^ allowing simultaneous use of all three primers with target ORFs available from the ORFeome collaboration^11^ and others and (3) Gateway (Life Technologies)-mediated recombineering of the mutant PCR product into compatible destination vectors. HiTMMoB permits simplified and sequential completion of mutagenesis and ORF cloning without intermediate DNA gel purification steps, making it cost-effective and scalable to a 96-well format leading to individual bacterial colonies carrying expression-ready mutant ORFs in less than 48 h. Shown in Supplementary Data 1 are representative ORF mutagenesis attempts (missense mutations and small insertions/deletions) for 175 reactions, where HiTMMoB achieved 99% mutation success (173 mutant ORFs constructed in a single attempt) to target ORFs ranging from 303 to 4,863 bp in length and 38 to 74% in DNA G/C content. In this example, HiTMMoB achieved 76% efficiency based on the number of mutation-positive ORFs isolated from multiple single bacterial colonies resulting from each mutagenesis reaction. The fact that HiTMMoB achieves ∼75% efficiency per reaction allows straightforward clonal isolation of individual ORFS, which we fully sequence-verify before use in downstream applications. For example, as shown in Supplementary Data 1, an average of 3.8 colonies per each of the 175 mutagenesis reactions was sufficient to obtain 173 of 175 desired mutant clones.

HiTMMoB also incorporates co-integration of individual DNA ‘BCs' with wild-type or mutant ORFs into destination vectors using multifragment DNA recombination (Fig. 1a). The barcoding strategy relies on a panel of individual BC entry clones, each designed to contain a unique 24-bp BC designed to be an isothermal set so that individual BCs are recombined into destination vectors only in the presence of wild-type or mutant ORFs. This flexible barcoding approach allows rapid use and recycling of BCs during ORF library preparation in diverse destination vectors, providing barcoded expression clones within 24 h following recombination, thus eliminating the need to BC entire ORF collections before use. This barcoding flexibility is especially useful when constructing allelic series of an individual gene (see Supplementary Data 1, for example), as each variant of the series can be simultaneously barcoded without any need of inserting BCs into every vector as typically done using conventional DNA ligation methods. Each BC serves as a surrogate identifier for its associated ORF, thus permitting individual ORF detection by NGS. Individual ORF enrichment (positive selection) or depletion (negative selection) among pooled ORFs can be determined by comparing the number of individual BC reads within experimental pools as a ratio of each BC to the total number of total BC reads per sequenced amplicon. For example, in the case of *in vivo* tumour driver enrichment screens (Fig. 1b) as used here, ORF-driven tumours (output) will be positively enriched for driver-associated BCs (BC5) and lose those with no role in progression (that is, passengers, BC1-4) compared with injected cells (input) carrying all barcoded ORFs.

### Model construction for PDAC driver discovery

**Figure 2:**
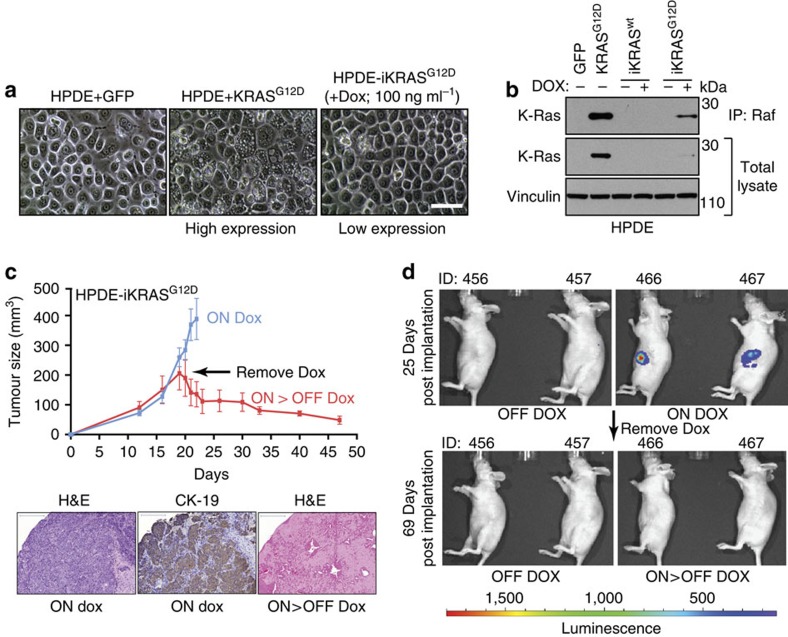
HPDE-*iKRAS*^*G12D*^ cell model. (**a**) HPDE cells expressing (left) GFP control and (middle) high levels of *KRAS*^*G12D*^. (Right) HPDE-*iKRAS*^*G12D*^ cells treated with Dox. Scale bar, 5 μm. (**b**) Immunoblot analysis of parental HPDE cells expressing GFP or high levels of *KRAS*^*G12D*^ (leftmost two lanes) and the indicated HPDE-inducible cell lines. Protein lysates were immunoprecipitated (IP) with antibody against RAF as an indicator of Ras activation. (**c**,**d**) HPDE-i*KRAS*^*G12D*^ xenograft growth (mean tumour volume, error bars denote s.d.) of biological replicates (*N*=7 bilaterally injected animals=14 tumours per group) in the presence of Dox (On Dox) and following removal of Dox (On>Off Dox) implanted subcutaneously (**c**) and orthotopically into the pancreas (**d**). Representative haematoxylin and eosine and CK-19 staining of the indicated tumours shown at bottom. Scale bars, 200 μm.

We first sought to apply HiTMMoB to identify novel, rare aberration drivers of PDAC. In an effort to model the genetic context of early PDAC, we integrated a doxycycline (Dox)-regulatable *KRAS*^*G12D*^ transgene (*iKRAS*^*G12D*^) into previously engineered non-transformed, human pancreatic ductal epithelial (HPDE) cells previously engineered^15^,^16^ with the E6 and E7 proteins of the HPV16 virus to emulate loss of p53 and inactivation of the Rb pathway, respectively^15^,^16^. Parental HPDE cells expressing high levels of *KRAS*^*G12D*^ underwent significant morphological changes and loss of proliferative capacity (Fig. 2a) consistent with oncogene-induced senescence^17^. In contrast, HPDE-*iKRAS*^*G12D*^ cells treated with Dox appeared to be morphologically similar to parental cells (Fig. 2a) owing to the titratable and near-physiological level of KRAS protein (Fig. 2b) that reduces the potential for *KRAS*^*G12D*^-mediated senescence. RAF-immunoprecipitation analysis (Fig. 2b) indicated a significant *KRAS* activity in HPDE-*iKRAS*^*G12D*^ cells treated with Dox compared with untreated and wild-type (HPDE-*iKRAS*^*wt*^) control cells. RNA profiling of HPDE-*iKRAS*^*G12D*^cells revealed widespread changes in the presence of Dox (2,824 gene probes; fold change >2.0 for on-Dox versus off-Dox, *P*<0.01, *t*-test) compared with HPDE*-iGFP* and -*iKRAS*^*wt*^ cells (Supplementary Fig. 1a and Supplementary Data 2). Within the *KRAS*^*G12D*^-induced genes, the Molecular Signatures Database identified ‘Bild_Hras_Oncogenic_Signature' as a top-enriched gene set (*P*=3.76 × 10^−29^, one-sided Fisher's exact test; Supplementary Data 3). Upregulation of Ras signalling in Dox-treated HPDE-*iKRAS*^*G12D*^ cells was also supported by a significant overlap of *KRAS* dependency signature genes reported by Settleman and colleagues^18^ (*P*=7.0E^−22^, one-sided Fisher's exact test) and Reverse-Phase Protein Array (RPPA) analysis (Supplementary Fig. 1b).

We next evaluated HPDE-*iKRAS*^*G12D*^ cells for oncogenic growth. Placing the cells in anchorage-independent growth conditions in the presence of Dox led to a significant (*P*=0.0001, *t*-test) increase in colony formation compared with off-Dox control cells (Supplementary Fig. 1c), and implanting HPDE-*iKRAS*^*G12D*^ cells subcutaneously into the flanks of athymic mice (*N*=14) led to a rapid formation of poorly differentiated, CK-19-positive carcinomas (*N*=12) in animals on Dox compared with no tumours observed in animals maintained off Dox (Fig. 2c). Randomizing on-Dox animals into an off-Dox cohort led to complete tumour regression, revealing remnants of pre-existing tumour with underlying vasculature in co-injected Matrigel (BD Biosciences) matrix (Fig. 2c). We next modified HPDE-*iKRAS*^*G12D*^ cells by including a constitutively expressing allele of firefly luciferase whose expression enables live animal serial imaging following injection of D-luciferin. Similar to the subcutaneous tumour growth studies above, HPDE-*iKRAS*^*G12D*^ cells progressed to tumours within 2 weeks post implantation into the pancreas of mice maintained on Dox diet, and these tumours regressed below the limits of detection within 45 days following removing Dox (Fig. 2d). In summary, we derived and characterized an HPDE-*iKRAS*^*G12D*^ cell model for use in primary screens to identify PDAC gene aberrations that drive *in vivo* tumour growth.

### *In vivo* pooled screening for aberration drivers of PDAC

We next resourced PDAC tumour sequencing data from the ICGC^13^, whose analyses by three separate statistical methods (Fisher's combined *P* value test, likelihood ratio test and convolution test) identified significantly mutated genes containing non-silent mutations. We first chose the 59 significantly mutated genes ranked in the top 100 by all three statistical methods following exclusion of known drivers *KRAS*, *TP53*, *CDKN2A*, *SMAD4*, *ATM* and *ARID1A* (Supplementary Table 1). For the remaining 53 gene candidates, we employed HiTMMoB to engineer 73 unique, non-silent mutations into 41 ORFs after excluding aberrations where ORFs were unavailable. In total, we were able to construct 70 (95.9%) barcoded aberration clones for entry into the functional screening platform.

**Figure 3:**
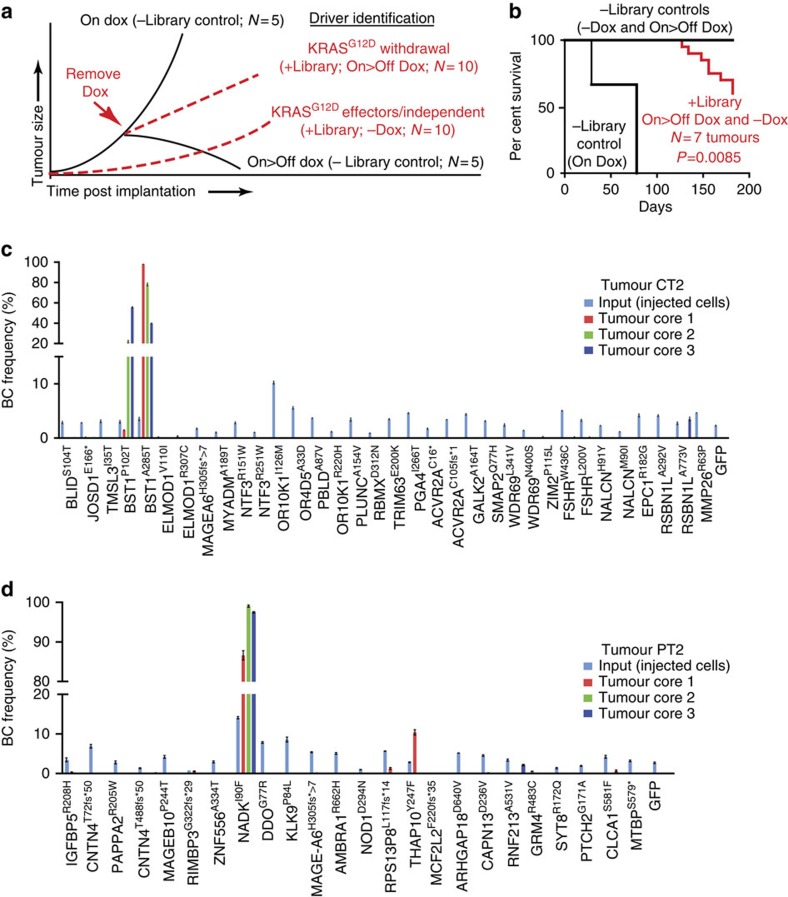
*In vivo* functional screening for aberration drivers of PDAC. (**a**) Screen schematic. HPDE-*iKRAS*^*G12D*^ cells expressing GFP only (−Library control; *N*=5 each for On Dox and On>Off Dox cohorts) or candidate-barcoded ORFs plus GFP were pooled (+Library; *N*=10 each for On Dox and On>Off Dox cohorts) and injected into athymic mice subcutaneously. Mice were divided into On Dox, Off Dox (−Dox) or transitioned On to Off Dox (ON>OFF Dox) cohorts to look for *KRAS*-dependent and *KRAS*-independent drivers. (**b**) Kaplan–Meier survival plot for computationally informed screen cohorts as indicated in **a**. *P* value calculated by log-rank test. (**c**,**d**) Barcode enrichment analysis of representative tumour (**c**) CT2 and (**d**) PT2. Data post normalization to total reads (sum of reads of barcodes+GFP) are shown as mean±s.d. of sequencing technical replicates (*N*=3 for each input and core).

To examine oncogenic activity of these computationally selected aberrations, HPDE-*iKRAS*^*G12D*^ cells stably transduced with barcoded control (green fluorescent protein (GFP)) and aberration lentivirus were pooled into two populations, each implanted into a single flank of 20 athymic mice (total 40 animals, Fig. 3a). For each of the two screen cohorts, 10 animals were maintained off Dox diet to identify drivers that function downstream or independent of *KRAS*^*G12D*^. The remaining 10 animals for each screen cohort were placed on Dox diet to promote *KRAS*^*G12D*^-driven tumorigenesis, followed by transitioning animals with palpable tumours (200 mm^3^) off-Dox (denoted On>Off Dox) to identify events that enable tumour escape from Dox withdrawal. All animals were monitored for tumour formation and growth as compared with reference control mice (Fig. 3a). Screening using this approach identified seven tumours (CT1–7; *N*=6 in mice off-dox, *N*=1 in mice On>Off Dox) 18–26 weeks post injection (Fig. 3b). To assess ORF enrichment in those seven tumours, we quantitated ORF BC reads from genomic DNA (gDNA) isolated from tumour (output) and injected cells (input) using an Ion Torrent Personal Genome Machine (PGM; 66 multiplexed samples: three input gDNA replicates + three gDNA replicates from three individual cores for each of the seven tumours; replicate=technical replicate PGM amplicon library preparations). Analysis of the 21 cores taken from CT1–7 revealed a strong enrichment for variants of *Bone Marrow Stromal Cell Antigen 1*, *BST1* (*BST1*^*P102T*^ and *BST1*^*A285T*^) versus input control (representative tumour CT2 shown in Fig. 3c and Supplementary Fig. 2). We observed minimal variation across technical replicate for amplicon PGM library preparations (see error bars, Fig. 3c). Notably, individual cores from CT6 and CT7 exhibited enrichment for *BST1* variants along with a second ORF (*WDR69*^*L341V*^; Supplementary Fig. 2), indicating an ability to identify multiple driver ORFs acting alone or in combination in a given tumour. Similarly, tumours CT5 and CT6, which demonstrated significant necrosis at the time of animal necropsy, thus increasing sequencing deviation across replicate PGM libraries, revealed high-level enrichment of *BST1*^*P102T*^ and *BST1*^*A285T*^ as well as co-enrichment for *ELMO/CED-12 Domain Containing 1*, *ELMOD1*^*R307C*^, in all tumour cores (Supplementary Fig. 2).

In addition to our computational approach towards candidate selection, we also took an unbiased approach by randomly choosing four patients sequenced by the ICGC^13^ whose tumours contained activating and inactivating mutations in *KRAS* and *TP53*, respectively. We then engineered aberration (missense mutations and small insertions/deletions; *N*=24) expression constructs for all events in the four patients in genes represented in our ORF collection (Supplementary Table 1). Screening using the same *in vivo* approach (Fig. 3a) identified four tumours (PT1–4) in the library-injected cohorts (*N*=3 in mice off Dox, *N*=1 in mice On>Off Dox) at 21 weeks post injection. BC enrichment analysis of 40 multiplexed samples (four replicates for Input gDNA + three gDNA replicates from three individual cores for each four tumours) revealed a high-level enrichment for mutant *NADK* (*NADK*^*I90F*^; average 94% enrichment) in three out of four tumours (PT2–4) as well as a low-level enrichment (11%) for *THAP Domain Containing 10*, *THAP10*^*Y247F*^, in a single core of PT2 (Fig. 3d and Supplementary Fig. 3). Notably, tumour PT4 demonstrated significant necrosis at the time of necropsy, and sequencing analysis revealed co-enrichment for *Insulin-Like Growth Factor Binding Protein 5*, *IGFBP5*^*R208H*^, in two cores (C1 and C2) taken from the necrotic region of the tumour compared with a non-necrotic core (C3) fully enriched for *NADK*^*I90F*^ (C1, 99%; Supplementary Fig. 3). Finally, analysis of PT1 (Supplementary Fig. 3) indicated complete enrichment of Melanoma Antigen Family A-6, *MAGE-A6*^*CΔ304*^, across all three cores analysed for this tumour. In summary, this first use of HiTMMoB for primary screening of ICGC data prioritized driver aberrations with greatest enrichment for variant ORFs encoding *BST1, MAGEA6* and *NADK* (Supplementary Table 2).

### *NADK* ^*I90F*^ exhibits GOF activity in PDAC

**Figure 4:**
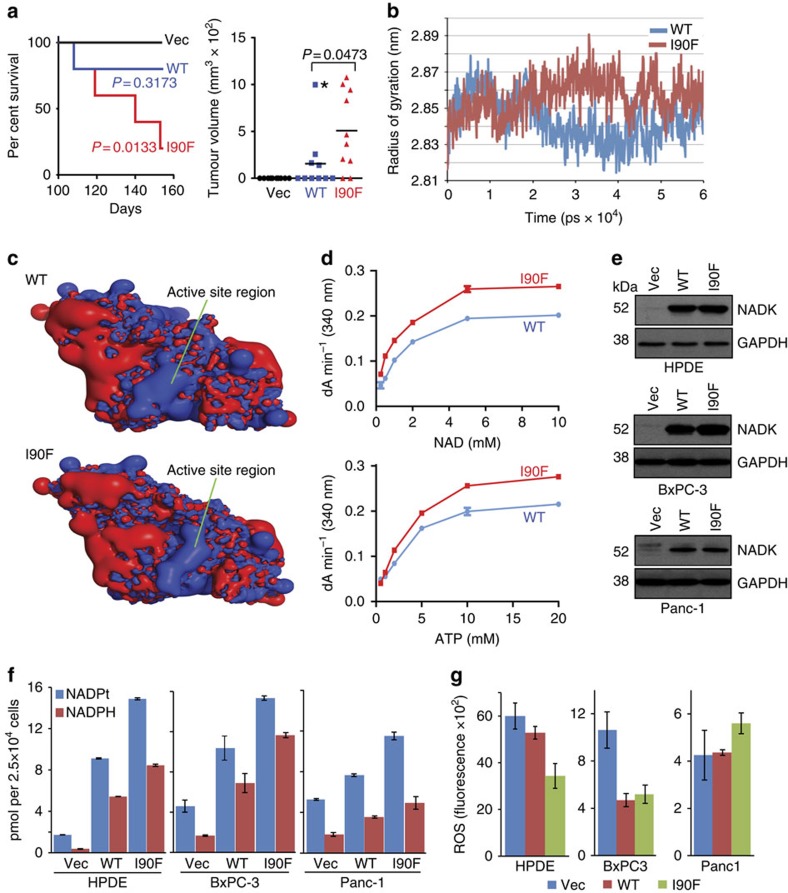
*NADK*^*I90F*^ promotes tumorigenesis and alters redox state. (**a**) Kaplan–Meier survival plot for xenograft assay of HPDE-*iKRAS*^*G12D*^ cells expressing GFP (Vec), *NADK*^*WT*^or *NADK*^*I90F*^ in mice maintained Off Dox diet (*N*=10 each). *P* value calculated by log-rank test. Complementary end point tumour volume shown at right, *P* value by *t*-test that includes *NADK*^*WT*^ outlier. *, outlier based on Grubb's extreme studentized deviate outlier test, *P*<0.05. (**b**) Protein radius of gyration plot. (**c**) Electrostatic potential plots comparison of wild type to I90F mutant (regions of negative potential are coloured red; regions of positive potential are coloured blue). (**d**) Reaction kinetics of NAD and ATP substrates for NADK WT and I90F mutant recombinant enzymes. Error bars denote s.d. of replicates (*N*=3). (**e**) Immunoblot analysis of NADK expression in the indicated cell lines used for (**f**) quantitation of cellular NADPt/NADPH (mean value±s.d. of replicates, *N*=3 each) and (**g**) ROS through measurement of DCFDA fluorescence (mean value±s.d. of replicates, *N*=3 each).

Our primary screen revealed high enrichment for a mutant ORF encoding *NADK* (*NADK*^*I90F*^), which was identified by the ICGC^13^ to be mutated in a single tumour (PCSI0023_T). NADK catalyses the conversion of cytoplasmic NAD^+^ to NADP^+^/NADPH and contributes to cellular NADPH production^19^. We chose to validate *NADK* driver activity, given the importance in maintaining the redox state in PDAC^20^, potential druggablity as a kinase and our premise that functionalizing rare mutations, even those unique to a single patient, may inform new modes to activate important signalling pathways. Re-construction and implantation of HPDE cells expressing *NADK*^*I90F*^ validated its tumour-initiating activity (*N*=8/10; *P*=0.0133, log-rank test) compared with vector control (*N*=0/10), leading to a significant decrease in overall survival for the *NADK*^*I90F*^ cohort (Fig. 4a). Wild-type *NADK* exhibited very weak tumour-initiating activity (*N*=4/10), as three of the four *NADK*^*WT*^ tumours remained growth-static or -regressed (Fig. 4a). In contrast, *NADK*^*I90F*^-expressing tumours grew to maximal tumour burden and were significantly larger than the static *NADK*^*WT*^-expressing tumours, despite a single *NADK*^*WT*^ outlier tumour (Fig. 4a; Grubb's extreme Studentized deviate outlier test, *P*<0.05).

*In silico* molecular dynamics simulation of the I90F mutation based on the crystal structure of dimerized human NADK^21^ indicated a significant I90F-induced change in the radius of gyration (Fig. 4b), a measure of structure compactness, predicted to have an impact on catalytic activity as observed in other mutant enzymes^22^. Brooks *et al*. observed that distal mutations can have an impact on correlated motions along the backbone of an enzyme, thereby having an impact on its catalytic activity^22^. Consistent with this finding, simulation trajectory analyses revealed an increase in coupled motions in the I90F variant compared with wild-type (Supplementary Fig. 4). Moreover, plotting the electrostatic potential of the substrate-binding region of NADK revealed a significant change in shape and size of the positive electrostatic potential in the I90F variant compared with wild type (Fig. 4c), suggesting that the I90F mutation opens the NADK dimer interface to increase substrate availability, leading to greater kinase activity. Together, these data led us to examine kinase activity by recombinant wild-type and I90F mutant protein (Supplementary Fig. 5). NAD+ and ATP enzymatic assays (Fig. 4d) revealed an overall increase in *in vitro* kinase activity of recombinant NADK^I90F^ (NAD+: *V*_max_, 0.29; *K*_m_, 0.91. ATP: *V*_max_, 0.33; *K*_m_, 03.28) compared with NADK^WT^ (NAD+: *V*_max_, 0.23; *K*_m_, 1.23. ATP: *V*_max_, 0.25; *K*_m_, 03.77). Together, these data indicate that the I90F mutation in NADK provides GOF activity, leading to an increase in tumorigenesis and tumour growth by HPDE cells.

Given NADK's known role in phosphorylating NAD, we next quantitated total (NADPt) and reduced (NADPH) NADP in cells expressing wild-type and mutant *NADK* (Fig. 4e). Analysis revealed a 23-fold increase in NADPH in HPDE cells expressing *NADK*^*I90F*^ compared with vector control cells, and the effects of expressing *NADK*^*I90F*^ were greater than wild-type (sixfold; mutant versus wild-type NADK, *P*=0.0007, *t*-test) consistent with GOF activity for the I90F mutation (Fig. 4f). We confirmed these results using two PDAC cell lines, BxPC-3 and Panc-1, both of which exhibited a similar trend in NADPt/NADPH levels (Fig. 4f). Consistent with increased NADPH levels, HPDE cells expressing *NADK*^*I90F*^ also exhibited less ROS compared with wild-type- and vector-expressing cells (Fig. 4g), suggesting that increased *NADK* activity elevates NADPH, resulting in a redox shift to protect cells against oxidative stress. Wild-type and mutant *NADK* similarly reduced ROS in BxPC-3 cells, but did not reduce ROS in Panc-1 cells, consistent with the modest increase in NADPH (Fig. 4f) and low ROS baseline level in this cell line (Fig. 4g).

### Wild-type *NADK* depletion attenuates PDAC growth

**Figure 5:**
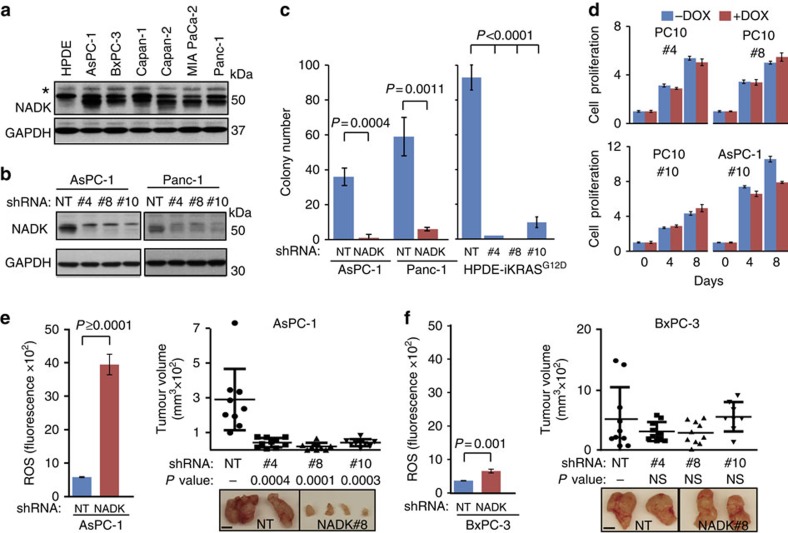
*NADK* knockdown promotes cellular ROS and abrogates PDAC growth. (**a**,**b**) Immunoblot analysis of endogenous NADK expression in (**a**) HPDE and the indicated PDAC cell lines and (**b**) in AsPC-1 and Panc-1 cells following RNAi-mediated depletion (shNADK#4, 8 and 10) versus NT control. * designates nonspecific band. (**c**) RNAi-treated cells in assessed for anchorage-independent growth. Shown are mean values±s.d. of replicates (*N*=3 each); *P* value calculated by *t*-test. HPDE*-iKRAS*^*G12D*^ cells were grown and plated in Dox. (**d**) Dox-inducible RNAi-treated PC10 fibroblasts and AcPC-1 cells assessed for proliferation in the presence or absence of Dox. Shown are mean values±s.d. of replicates (*N*=4 each). Full growth curves in Supplementary Fig. 6. (**e**,**f**) Impact of *NADK* depletion on cellular ROS (mean values±s.d. of replicates, *N*=3 each) and xenograft tumour growth (*N*=10 each) in (**e**) AsPC-1 and (**f**) BxPC-3 cells. The mean tumour volumes denoted as horizontal bars, s.d. range denoted by vertical T bars. *P*-values calculated by *t*-test.

The I90 residue is adjacent to the NADK kinase domain, and missense mutations in *NADK* have been observed across other cancer types at low frequency based on mining cBioPortal (http://www.cbioportal.org/public-portal). The fact that cancer genes are often deregulated by multiple genetic and epigenetic mechanisms led us to examine expression of wild-type *NADK*. Consistent with this notion, immunoblot analysis of a PDAC cell line panel revealed a moderate- to high-level expression of *NADK* compared with primary cells (Fig. 5a). To determine whether wild-type *NADK* is required for PDAC cell growth, we identified three short hairpin RNAs (shRNAs; shNADK#4, 8 and 10) whose lentiviral delivery to high *NADK*-expressing cell lines AsPC-1 and Panc-1 significantly depleted NADK protein expression compared with non-targeting (NT) control (Fig. 5b). Functional assessment of *NADK* knockdown in AsPC-1 and Panc-1 cells revealed a significant impact on anchorage-independent growth in both cell lines (Fig. 5c; 36.0- and 10.4-fold decrease, respectively), which we confirmed in HPDE-*iKRAS*^*G12D*^ cells grown in the presence of Dox (Fig. 5c; *P*<0.0001, *t*-test), thus indicating an ability of *NADK* repression to significantly abrogate *KRAS*^G12D^-driven proliferation. Depletion of *NADK* in normal fibroblasts (PC10) using the same three shRNAs (shNADK#4, 8 and 10) cloned into a Dox-inducible shRNA expression vector^23^ had no effect on proliferation in the presence of Dox compared with off Dox (Fig. 5d and Supplementary Fig. 6a), whereas inducible knockdown of *NADK* had a marked effect on AsPC-1 cancer cell proliferation by 8 days following Dox addition (Fig. 5d and Supplementary Fig. 6b) consistent with growth in soft agar (Fig. 5c). Knockdown of *NADK* in AsPC-1 cells also led to a marked 6.8-fold (*P*<0.0001, *t*-test) increase in cellular ROS levels versus NT control (Fig. 5e). Importantly, this sharp increase in ROS on *NADK* depletion corresponded with a negative impact on AsPC-1 xenograft tumour growth (Fig. 5e; *P* value=0.0003, *t*-test, averaged across three shRNAs versus NT), suggesting that elevated levels of ROS may be inhibitory to PDAC growth as reported previously (ref. 24 and reviewed in ref. 25). Consistent with this precept, knockdown of *NADK* in BxPC-3 cells led to a modest 1.8-fold increase in ROS compared with NT and did not significantly have an impact on BxPC-3 xenograft growth (Fig. 5f). Together, these data combined with the recognized importance of maintaining a redox state for PDAC tumour growth nominate *NADK* for additional target biology aimed at establishing its role as a novel target for drug development.

## Discussion

In the era of genomics medicine, we are faced with the daunting challenge of translating genome-scale knowledge to patient care. For cancer, therapeutically targeting oncoproteins or their effectors offer great hope for improving patient outcomes. However, the use of sequencing data for drug development or re-positioning of FDA-approved drugs is challenging since bioinformatic annotation alone is insufficient to inform such efforts. The research community should therefore direct its resources towards the most promising driver candidates, identification of which requires robust pipelines that allow functional prioritization of the thousands of potential targets emerging from sequencing data. We describe here the infrastructure that enables functionalization of somatic mutant gene variants through their efficient construction, molecular barcoding and delivery to cancer models for *in vivo* tumour driver screens. While also useful for the study of low-frequency, uncharacterized tail mutations in known cancer genes, here we focus on rare mutations in genes with less or no characterized roles in cancer. Even though mutated at low frequency, the biological selection of rare mutations during malignancy, detection of activity during functional screening and initial insights into their mode-of-action may reveal new mechanisms by which tumours activate known or novel cancer signalling pathways. Much like previous efforts towards functionalizing high-frequency aberrations for clinical translation (that is, targeted therapeutics for activating mutations in *EGFR*, *BRAF* and others), identifying rare driver mutations and their mode-of-action has the potential to reveal new tumour vulnerabilities, particularly when found to activate pathways with existing therapies.

We first applied these technologies to functionally annotate PDAC gene aberrations, work that prioritized *NADK*^*I90F*^ for downstream functional studies and target validation. While identified in only a single PDAC patient, the mutant's GOF activity led us to investigate the role of wild-type *NADK*, which we show is expressed at high levels in PDAC cell lines compared with normal cells and regulates redox states known to be critical for PDAC progression. Despite the importance of NADPH on redox regulation, NADK has been largely understudied in cancer and only recently been explored as a therapeutic target^26^. The redox state in a cell is primarily determined by the balance of NADP+/NADPH to maintain pools of reduced glutathione, a key pathway to reduce ROS^27^. ROS, which have both pro- and anti-tumorigenic effects, are often higher in cancer compared with normal cells because of mitochondrial damage, increased metabolic rates or elevated expression of oxidizing enzymes^27^,^28^,^29^. A number of studies have suggested that PDAC cells prefer low ROS (reviewed in ref. 25), rationalized based on the dual nature of ROS where low ROS levels may be growth-promoting, while high levels are cytotoxic. This notion is further supported by recent work^24^ reporting a novel mechanism by which *KRAS* rewires PDAC tumours to maximize energy production and promote NADPH accumulation to maintain redox state and tumour growth. This non-canonical metabolic pathway, which thus far is unique to PDAC, results in a shuttling of glutamine-derived aspartate from the mitochondria to the cytoplasm via the *GOT1* transaminase to fuel NADPH production and decrease cellular ROS^24^. These findings along with our data lead us to hypothesize that activating *NADK* through mutation or increased expression may provide PDAC tumours an additional means to increase cellular NADPH, and the interplay between *GOT1*-mediated glutamine utilization and NADK activity may cooperatively influence redox state and tumour growth in PDAC. More work will need to be performed to examine the possible intersection between *GOT1* and *NADK*, as well as studies into the potential clinical impact of inhibiting these pathways for PDAC patient care.

In addition to *NADK*, our efforts revealed potential driver activity for variants of *BST1*, *MAGEA6*, *ELMOD1*, *WDR69*, *THAP10* and *IGFBP5*. Of these, *BST1* and *MAGEA6* variants exhibited robust enrichment across multiple screen tumour cores, suggesting that they exhibit potent tumour-driving activity. While additional work is needed to validate these candidate oncogenes, including assessing variant activity compared with wild type, *MAGEA6* and *BST1* have both been previously implicated in cancer. A recent study^30^ reported that *MAGEA6* is overexpressed in a variety of cancers that include breast and colon, and high *MAGEA6* expression in lung squamous cell carcinoma correlates with poor overall survival. Moreover, depletion of *MAGEA6* in colon and lung cancer cell lines abrogated clonogenic survival, whereas exogenous *MAGEA6* expression transformed primary colonic epithelial cells^30^ and promoted tumour growth and metastasis in xenograft models of thyroid cancer^31^. BST1 metabolizes NAD^+^ to produce cyclic ADP ribose and subsequently ADP-ribose^32^,^33^, indicating a possible role for BST1 in Ca^2+^ homeostasis and calcium signalling^34^. Notably, in monocytes the interaction between BST1 and the ECM initiates signalling cascade mediated by focal adhesion kinase and Src, leading to activation of the MAP Kinase and PI3K/Akt pathways commonly dysregulated in cancer^35^. Consistent with *BST1*'s potential role in cancer, it is overexpressed in ovarian tumours and is correlated with shorter survival^36^.

Future work is required to validate the activity and clinical significance of the mutations and genes identified in this study. Our focus on low-frequency driver mutations precludes identifying tumour cell lines carrying such mutations, which is particularly true for PDAC because of limited numbers of cell lines present throughout the research community; thus, it is currently not possible to determine whether silencing or complementing the described mutations would alter tumour growth by tumours/tumour cell lines harbouring those events. Future work should include engineering the identified point mutations into mice for crossing on PDAC mouse models^37^ driven by inducible *KRAS*^*G12D*^. Such models would permit genetic tractability (that is, presence or absence of *KRAS*^*G12D*^, *TP53* and so on) and the proper microenvironment for assessing each mutation's ability to promote transformation, tumour initiation and progression. Nevertheless, our HiTMMoB-based screening approaches succeeded in prioritizing these events for mechanistic evaluation and secondary target biology studies to assess suitability for drug or pathway inhibitor development. When applied more broadly to other cancer types and model systems, these GOF screening technologies will reveal the highest priority targets to expedite translation of genomics data into actionable diagnostic and drug development end points.

## Methods

### Cell culture

All cell lines were propagated at 37 °C and 5% CO_2_ in humidified atmosphere. HPDE^15^,^16^ cells were cultured in KSFM medium (Life Technologies). Panc-1, AsPC-1, Capan-1, Capan-2, MIA PaCa-2 and BxPC-3 were purchased from American Type Culture Collection and were cultured following their recommendations. PC10 cells (provided by Dr. Penelope Bonnen, Baylor College of Medicine) were cultured in RPMI+10% fetal bovine serum (FBS). All human cell lines were authenticated by fingerprinting using short tandem repeat testing where possible and were verified to be free of mycoplasma contamination before use. In some experiments as indicated, cells were propagated in Dox (100 ng ml^−1^) or glucose and glutamine-free DMEM supplemented with physiological plasma concentrations of 5 mM glucose and 0.5 mM L-glutamine (Corning). The Dox-regulatable *KRAS*^*G12D*^ HPDE cell line was constructed using the pInducer system^23^.

### Immunoblot analysis

Cells were lysed using RIPA buffer (Sigma) containing Protease Inhibitor Cocktail (Sigma) with phosphatase inhibitors (Calbiochem) for separation and blotting on polyvinylidene difluoride (Millipore). The following antibodies were used for immunoblotting: NADK (Santa Cruz; sc-100347); GAPDH (Santa Cruz; sc-25778); Vinculin (Santa Cruz; sc-25336), phospho-ERK1/2 (Cell Signaling; 9101 S) and KRAS (Santa Cruz; sc-30). All antibodies were diluted to 1:1,000 in 1% BSA. Full immunoblot scans are shown in Supplementary Figs 7 and 8. KRAS activity assay was performed by RAF pulldown using a Ras Activation Assay Kit (EMD-Millipore) by using 650 μg of cell lysate and 10 μg of RAF beads per reaction following the manufacturer's protocol.

### Gene expression and RPPA analyses

RNAs extracted from the indicated cell lines cultured with or without Dox (100 ng ml^−1^ for 72 h) were processed for hybridization on Agilent arrays (G3 Human GE 8x60K) by the Baylor College of Medicine Genome Profiling Core Facility. Array data were deposited in NCBI Gene Expression Omnibus (GEO) database (http://www.ncbi.nlm.nih.gov/geo/) under accession number GSE58055. RPPA was performed by the MD Anderson RPPA Core Facility using protein lysates extracted from the indicated cell lines. For each RPPA replicate, cells were washed with cold PBS twice and lysed with 100 μl lysis buffer (1% Triton X-100, 50 mM HEPES, pH 7.4, 150 mM NaCl, 1.5 mM MgCl_2_, 1 mM EGTA, 100 mM NaF, 10 mM Na pyrophosphate, 1 mM Na_3_VO_4_ and 10% glycerol, containing freshly added protease and phosphatase inhibitors from Roche Applied Science Cat. # 05056489001 and 04906837001, respectively), mixed with 4 × SDS sample buffer (40% Glycerol, 8% SDS, 0.25 M Tris-HCl, pH 6.8) and boiled for 5 min before RPPA. Two-sided homoscedastic *t*-tests (using log-transformed data) and fold changes were used to determine differentially expressed genes and proteins. Expression patterns were visualized as heatmaps using JavaTreeView. MSigDB (version 3.0) gene sets were searched using SigTerms^38^.

### HiTMMoB and functional screening

Site-directed mutagenesis was based on modified application of megaprimer PCR^39^. ORF entry clones used in this study (Supplementary Table 1) were from Life Technologies or the ORFeome 8.1 (http://horfdb.dfci.harvard.edu/). Ten nanograms of ORF DNA template was entered into a PCR reaction^14^ using 0.6 μM modified M13F (5′-GTAAAACGACGGCC-3′) and M13R (5′-AACAGCTATGACC-3′) primers and 0.08 μM mutation-specific primer (Supplementary Table 1) using KOD Hot Start DNA Polymerase (Novagen). Amplification parameters were as follows: 1 × [95 °C-4′]; 6 × {6 × [95 °C−45”, 72 °C-1'+30” per 1 kb length of ORF, 95 °C-45”']; 3 × [88 °C-45”, 42 °C-1', 72 °C-30” per 1 kb length of ORF, 72 °C-2']}. PCR reactions were treated with 1 μl DpnI before recombination into destination vector pLenti6 V5/Dest (Life Technologies) following the manufacturer's recommendations. For barcoding, a set of BC entry clones was constructed by annealing *in vitro* complimentary DNA oligonucleotides containing 24 nt BCs flanked by T3/T7 sequences and attB2r/B4 sites. Annealed oligonucleotides were subsequently recombined into modified pDONR223 by BP recombination, leading to entry clones whose BCs were flanked with attR2/L4 for multifragment recombineering (with ORF entry clone or mutagenesis PCR product) into pLenti6 V5/Dest BC that contains attR1 and attR4 recombination sites. STBL3 (Life Technologies) bacteria were heat-shock-transformed with the BP/LR reaction mixtures for vector propagation. Lentivirus was produced using standard virus packaging vectors and virus protocols. For HPDE (and their derivatives) transduction, cells were infected once and allowed to recover in supplemented KSFM medium for 48 h. For *in vivo* screening, 2 × 10^4^ cells per virally infected HPDE line and GFP control were mixed and re-suspended in a 1:1 solution of Hank's balanced salts (Invitrogen) and Matrigel (BD Bioscience) for subcutaneous implantation into female nude animals (Harlan, Indianapolis, IN) at 5 × 10^5^ total cells per site.

### BC sequencing

gDNA was extracted from library-infected HPDE-*iKRAS*^*G12D*^ cells (injected or Input) and three individual tumour cores (output) for quadruplicate and triplicate, respectively, PCR reactions to amplify the BC pools present within each sample using Platinum Super Mix (Life Technologies) and flanking primers common to each BC: 5′-CAATTAACCCTCACTAAAGG-3′ and 5′-CCGCCACTGTGCTGGATA-3′). Amplification parameters were as follows: 1 × [94 °C-4']; 35 × [94 °C-1', 54 °C-1', 68-1']; 1 × [68 °C-10']. Each replicate PCR amplicon (178 nt) was purified for PGM library preparation, where each amplicon was ligated to unique Ion Xpress Barcode Adaptors for PGM sequencing (318 V2 Chip) following the manufacturer's recommendations. Raw data were concatenated into one ‘reference' file and indexed using burrows-wheeler alignment tool^40^ for alignment of BC sequences (with parameters ‘-l7 -t12 -N -n3') for counting the occurrence of each BC. BC enrichment was assessed by quantitating the number of occurrences for each BC sequence as a ratio to total number of BC reads in each sample. S.d.'s were calculated for quadruplicate and triplicate reactions for input and output samples, respectively, and were plotted as error bars on the BC enrichment graphs.

### Cell assays

Soft agar colony formation assays were performed on six-well plates in triplicate. For HPDE-i*KRAS* and NADK shRNA-transduced cells, cells were pretreated with/without DOX (100 ng ml^−1^) for 24 h and were mixed thoroughly in cell growth medium containing 0.4% SeaKem LE agarose (Fisher) in KSFM±DOX, followed by plating on bottom agarose prepared with 0.7% agarose in KSFM±DOX. Each well was allowed to solidify and subsequently covered in 2 ml KSFM±DOX, which was refreshed every 3 days. Colonies were stained with 0.05% (wt/vol) iodonitrotetrazolium chloride (Sigma) and scanned at 1,200 d.p.i. using a flatbed scanner. For AsPC-1 and Panc-1, RPMI+FBS and DMEM+FBS were used instead of KSFM, respectively, followed by plating 3,000 cells in each well for each cell line as described above. For cell proliferation assays, transduced AsPC-1 and PC10 cells were pretreated with DOX (2 μg ml^−1^) for 48 h, followed by plating 2,000 and 1,000 cells on White Opaque 96-well microplates in quadruplicate, respectively. Cell density was assayed with CellTiter-Glo (Promega) using a Wallac Victor2 Multilabel Counter at multiple time intervals. All data were assessed by two-tailed *t*-test calculation using Prism 4 (Graphpad).

### Animal studies

Animal studies were conducted in accordance to our IACUC-approved protocol at the Baylor College of Medicine. For pancreas orthotopic injection, 1 × 10^6^ HPDE-i*KRAS*^*G12D*^ cells were injected with Matrigel (BD Biosciences; 1:1 ratio; 20 μl total volume) into the tail of the pancreas in athymic mice. Mice were put On or Off Dox according to experimental design. D-luciferin (Gold Biotechnology; 1 g per 66 ml; 100 μl per mouse) was injected via the retro-orbital sinus. Mice were placed into xenogeny machine and subjected to imaging within 5 min for 1 min. For xenograft tumour assays, HPDE-*iKRAS*^*G12D*^ cells transduced with GFP or ORF lentivirus (in pLenti6.3 backbone) or shRNA-treated cells were subcutaneously injected (1 × 10^6^ cells each site) into the flanks of female nude animals (athymic nude mice; CrTac:NCr-*Foxn1*^*nu*^ Harlan, Indianapolis, IN) following suspension in Hank's buffer at 1:1 with Matrigel (BD Biosciences). Animals were maintained On or Off Dox diet (2 g kg^−1^; BioServ). Tissue sections were stained with haematoxylin and eosine and in some cases with CK-19 (16858-1-AP, Proteintech). The slides were scanned using Pannoramic 250 Flash Whole Slide Digital Scanner (3DHISTECH).

### NADPt/NADPH and ROS quantification assays

NADPt/NADPH ratios were determined using a NADP/NADPH assay kit (Abcam; ab65349) as detailed in the manufacturer's protocol. Briefly, 3 × 10^6^ transduced HPDE and BxPC-3 cells and 5 × 10^6^ Panc-1 cells were plated on 10-cm plates for 24 h, followed by replacing growth medium by nutrient-deficient DMEM (Corning; 17-207-CV)+10% FBS+5 mM glucose+0.5 mM glutamine for 6 h before harvest. Overall, 2 × 10^6^ cells were collected and immediately used for sample detection by and absorbance measurement using a Wallac Victor2 Multilabel Counter. For ROS measurements, 3.5 × 10^5^ transduced HPDE and BxPC-3 cells and 5 × 10^5^ Panc-1 and AsPC-1 cells were plated on six-well plate in triplicate for 24 h, followed by replacing growth medium by nutrient-deficient DMEM (Corning; 17-207-CV)+10% FBS+5 mM glucose+0.5 mM glutamine for 6 h before harvest. Cells were stained with 5 μM 2′,7′-dichlorodihydrofluorescein diacetate (DCFDA) (Invitrogen) for 30 min before analysis using flow cytometry according to the manufacturer's protocol.

### NADK modelling of mutant I90F

Models were built from the 3PFN.pdb (ref. 21) X-ray crystal structure of human NADK and chains A and B only (the dimer). We used the UCSF Chimera^41^ DockPrep tool to replace the selenomethionine (MSE) residue with methionine (MET) and replace all missing side chains in the structure using Dunbrack rotamer library^42^. The ATP and Mg^2+^ ions were docked manually based on least squares fit of the protein backbones with the X-ray crystal structure of NADK from *Archaeoglobus fulgidus* in complex with ATP^43^. We used SPDBV (Swiss PDB viewer known as ‘DeepView'; ref. 44) to make the I90F mutant. All calculations were performed using the Gromacs v 4.6.5 biomolecular dynamics software suite^45^. We used the Amber99SB-ILDN force field^46^ for the protein and the general amber force field (47) for the ATP molecule. The models were refined using steepest-descent energy minimization for both *in vacuo* structures using a 12-Å cutoff with a shift function to smoothly decay forces from 10 to 12 Å to a gradient 1000, kJ mol^−1^·nm. This method was followed by conjugating gradient minimization to a gradient of 100 kJ mol^−1^·nm. Each system was solvated in a periodic octahedral box of TIP3P water^48^, with 0.15 M NaCl giving a neutral system. The water was energy-minimized using steepest descents to a gradient of 1,000 kJ mol^−1^·nm using 10 Å nonbonded force cutoff and particle mesh Ewald (PME) method^49^,^50^ for long-range electrostatic interactions. We ran our simulations using a Tesla GPU card. The verlet cutoff scheme was used for all molecular dynamics simulations with a 9.0-Å short-range cutoff. The PME method was used to account for long-range electrostatic interactions with a PME order of 6. A dispersion correction for the energy and pressure was used to account for the use of a cutoff for the van der Waals interactions. The LINCS method^51^ was used to constrain bonds to hydrogen atoms to enable the use of a 2-fs time step for the simulations. The velocity rescale method^52^ was used to maintain simulation temperature to 300 K. The Parrinello–Rahman barostat^53^ was used to maintain pressure to 1 bar for the simulations. The following procedure was used: 100 ps Canonical ensemble (NVT) keeping protein restrained—>100 ps Isothermal-isobaric (NPT) ensemble keeping protein restrained—>60 ns of production molecular dynamics using NPT ensemble with no restraints. Structure averages were subjected to the same energy minimization refinement protocol as the initial models. Electrostatic calculations were performed using the APBS v 1.2.1 programme^54^ using *ɛ* (solute)=2.0; *ɛ* (solvent)=78.0; probe radius=1.4 Å; [NaCl]=0.15 M and *T*=310 K. Amber 99SB partial atomic charges and Amber atomic radii were used for the electrostatic calculations. We used the Pymol graphics package for all illustrations.

### *NADK* enzymatic assay

GST-NADK WT and GST-NADK^I90F^ were cloned into pGEX-6 P-1 and expressed in One Shot BL21 (DE3; Life Technologies) for purification using Glutathione Agarose (Thermo Scientific) following the standard protocol. *NADK* and *NADK*^*I90F*^ activity was measured spectrophotometrically with a coupled assay that reduces NADP generated by NADK into detectable NADPH using the enzyme glucose-6-phosphate dehydrogenase^55^. Data were analysed using GraphPad Prism 6 by means of Michaelis–Menten kinetics^56^.

### *NADK* knockdown studies

*NADK* knockdown studies were performed using pLKO-shGFP (NT) or each of the following shRNAs available from the RNAi Consortium (TRC; Broad Institute): TRCN0000037702 (shNADK#4), TRCN0000199808 (shNADK#8), TRCN0000199040 (shNADK#10). shRNA target sequences are as follows: shGFP (NT): 5′-CAAGCTGACCCTGAAGTTCAT-3′; shNADK#4: 5′-GCATCAGCATCACTACCTCAT-3′; shNADK#8: 5′-GATGACATTTCCAATCAGATA-3′; shNADK#10: 5′-GAATAAGGAATTGAGTCCAGA-3′.

## Additional information

**Accession codes:** The microarray data have been deposited in the NCBI GEO database under accession code GSE58055.

**How to cite this article:** Tsang, Y. H. *et al*. Functional annotation of rare gene aberration drivers of pancreatic cancer. *Nat. Commun.* 7:10500 doi: 10.1038/ncomms10500 (2016).

## Supplementary Material

Supplementary Information Supplementary Figures 1-8 and Supplementary Tables 1-2.

Supplementary Data 1 Representative HiTMMoB data.

Supplementary Data 2 HPDE-iKRAS gene expression analysis.

Supplementary Data 3 Molecular Signatures Database Analysis.
