# Supplementary material for: Functional annotation of rare gene aberration drivers of pancreatic cancer
Source: Nat Commun. 2016 Jan 25;7:10500. doi: 10.1038/ncomms10500 (PMC4737758; doi:10.1038/ncomms10500)
Supplement: Supplementary Information — Supplementary Figures 1-8 and Supplementary Tables 1-2. [file ncomms10500-s1.pdf]

Supplementary Figure 1

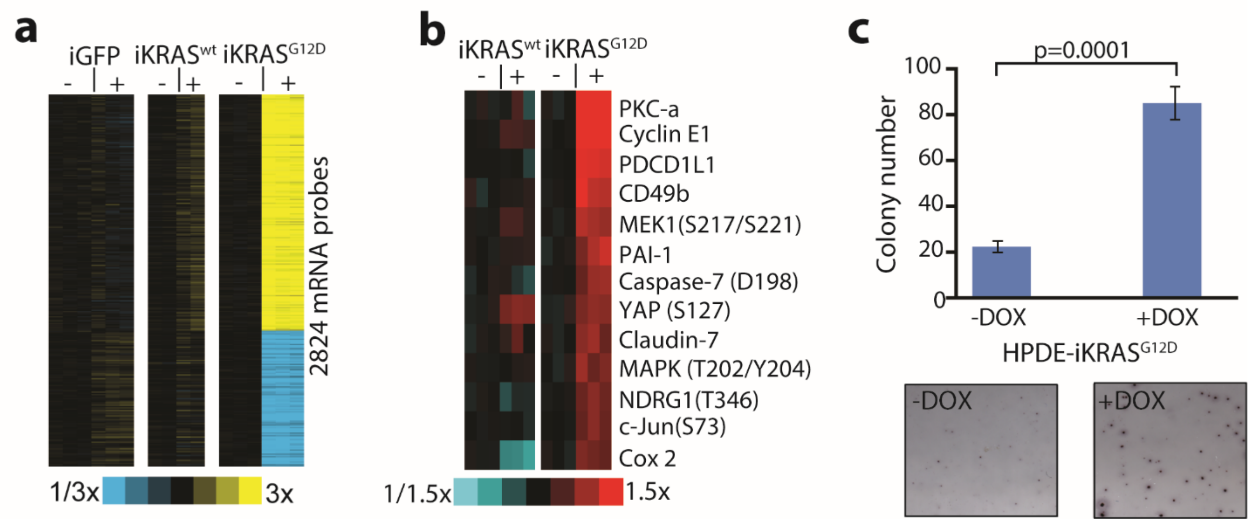

Supplementary Figure 1. HPDE-*iKRAS*^{G12D} characterization. (**a**-**b**) Differentially expressed (***a***) mRNAs ($p<0.01$, fold>2.0) and (**b**) proteins ($p<0.01$, fold>1.2) determined by gene expression profiling and RPPA, respectively, from the indicated cell lines. (**c**) HPDE-i*KRAS*^{G12D} cells assessed for anchorage-independent growth of biological replicates (N=3). Error bars denote standard deviation; p-value calculated by t-test.

Supplementary Figure 2

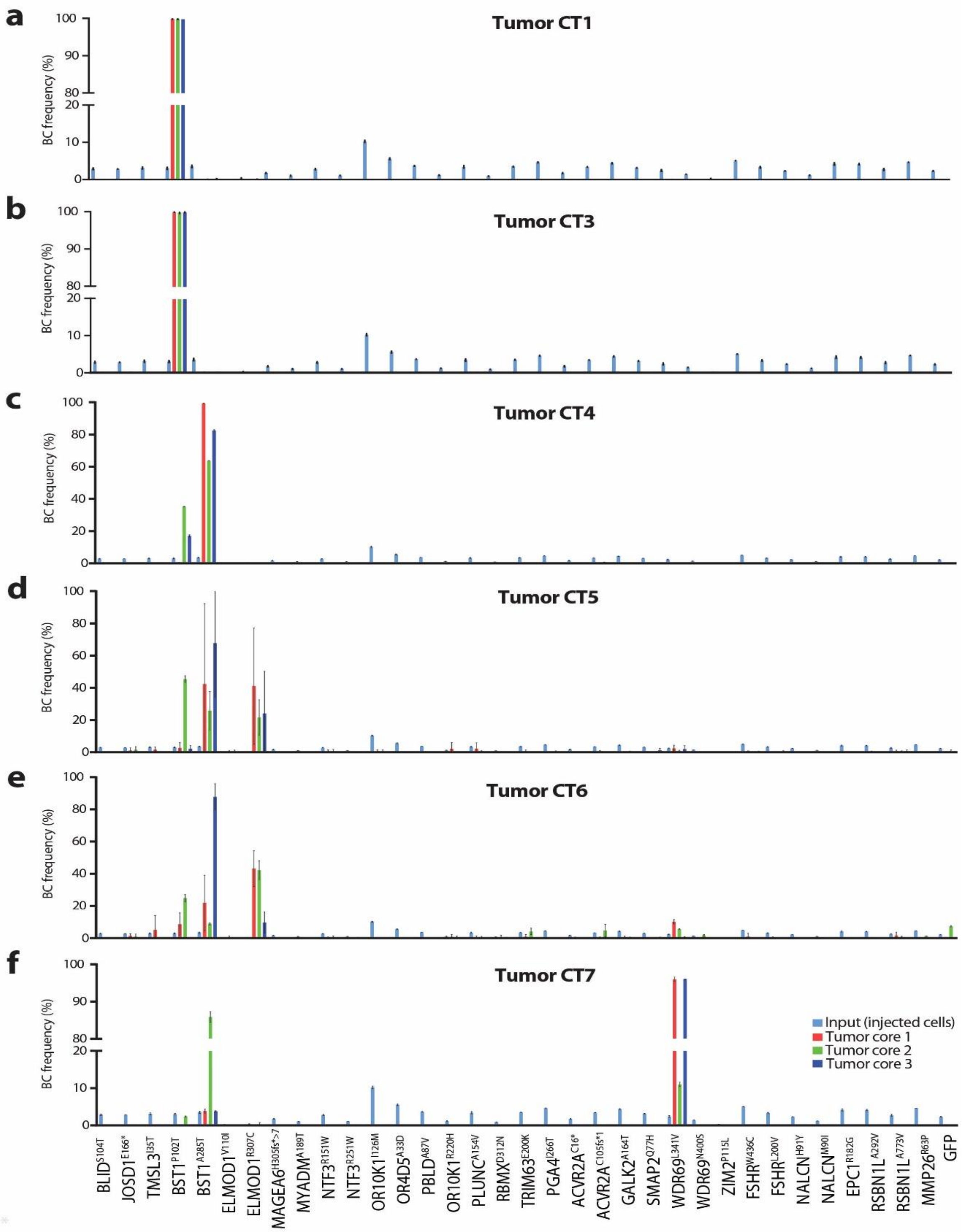

Supplementary Figure 2. Barcode enrichment analysis (CT tumors 1, 3-7). (**a**-**f**) Barcode enrichment analysis of tumors (**a**) CT1, (**b**), CT3 and (**c**) CT4, (**d**) CT5, (**e**) CT6, (**f**) CT7 resulting from computationally-selected library screening. Data post-normalization to total reads (sum of reads of barcodes + GFP) is shown as mean ± standard deviation (error bars) of sequencing technical replicates (N=3) per input and core samples.

Supplementary Figure 3

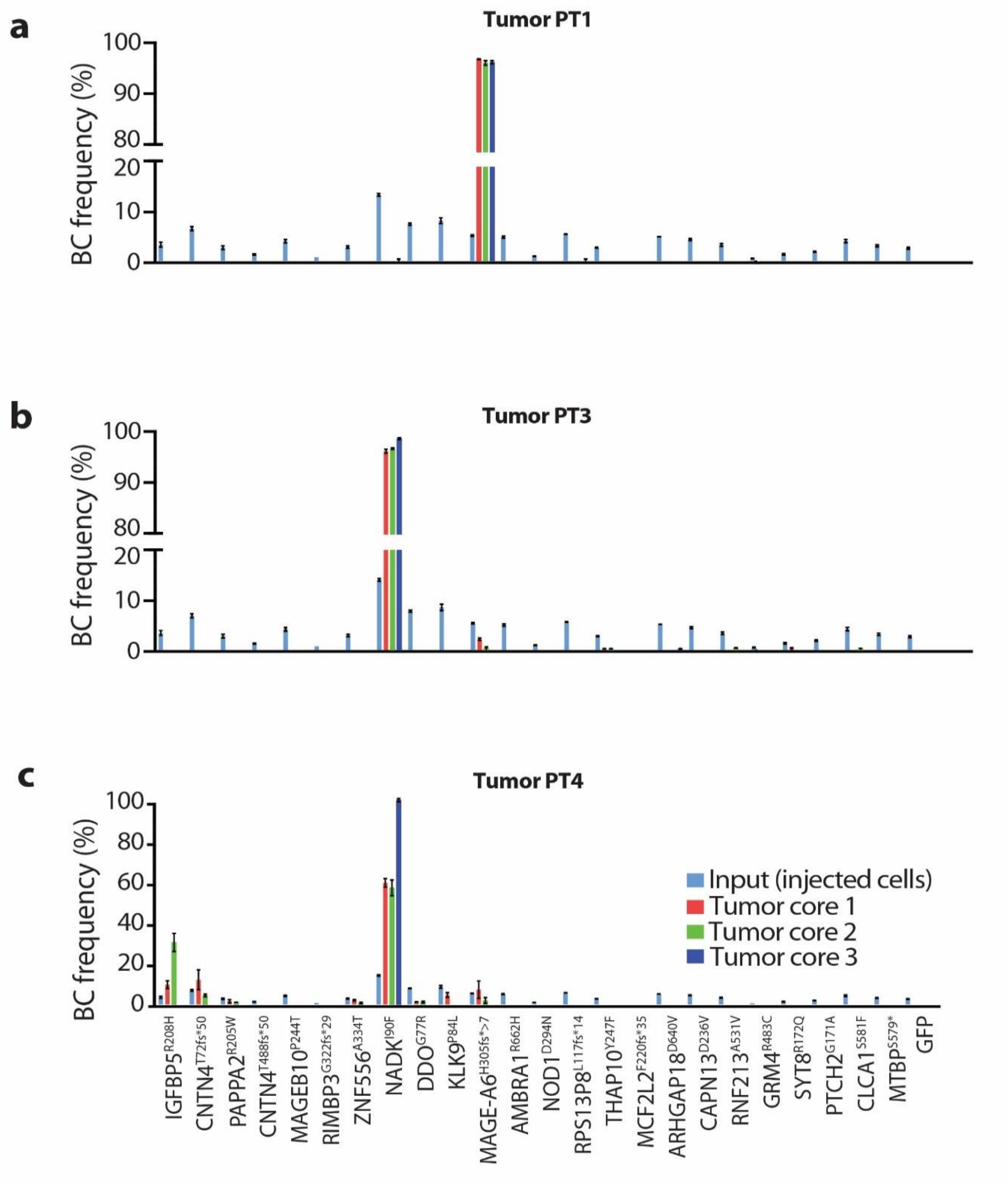

Supplementary Figure 3. Barcode enrichment analysis (PT tumors 1, 3-4). (**a-c**) Barcode enrichment analysis of tumors (**a**) PT1, (**b**), PT3 and (**c**) PT4 resulting from personalized functionalization library screening. Data post-normalization to total reads (sum of reads of barcodes + GFP) is shown as mean ± standard deviation (error bars) of sequencing technical replicates (N=3) per input and core samples.

Supplementary Figure 4

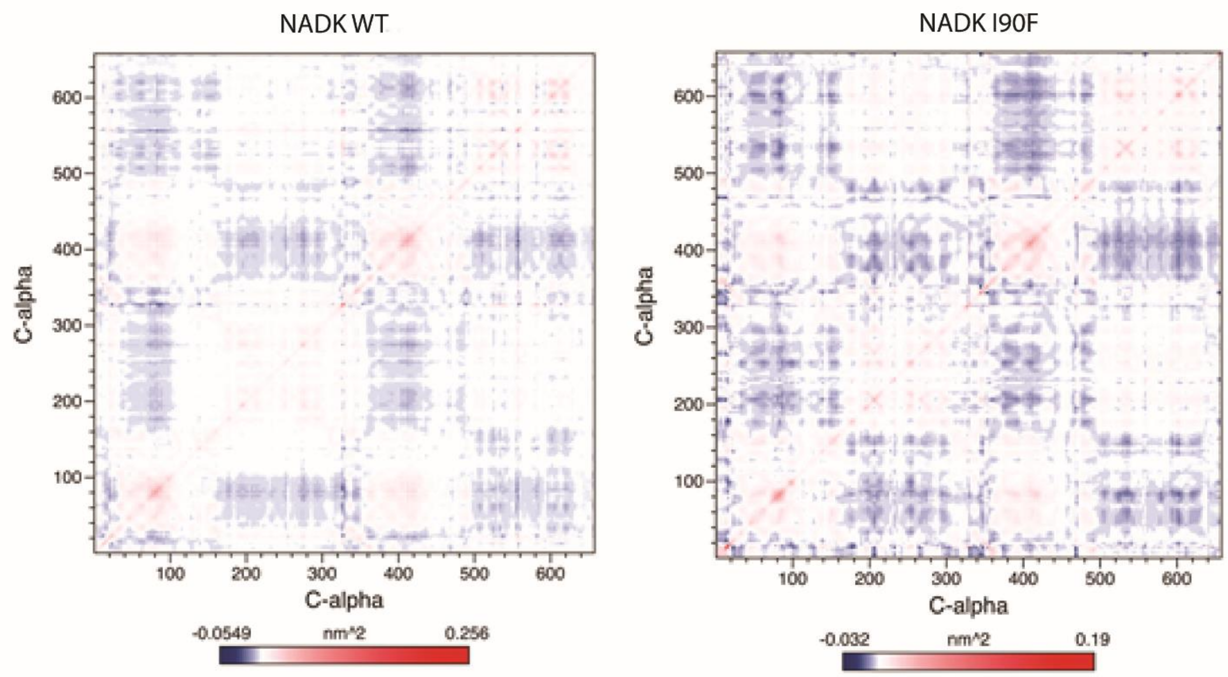

Supplementary Figure 4. Covariance matrix for the fluctuation of protein backbone C-alpha atoms. Red indicates correlated motions and blue indicates anti-correlated motions.

Supplementary Figure 5

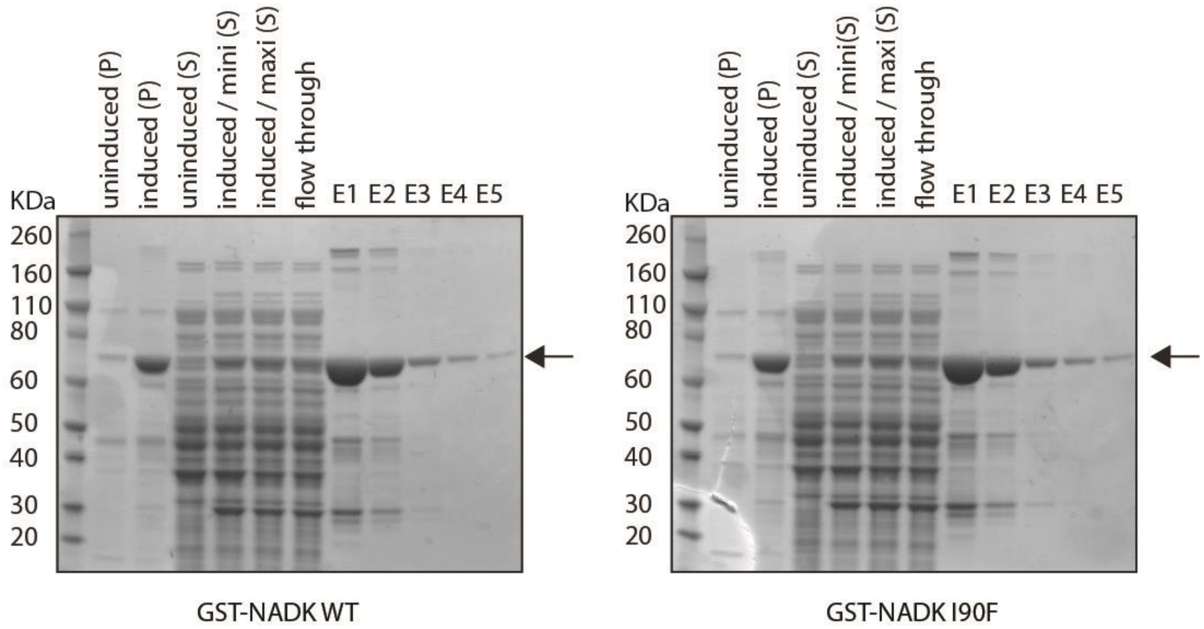

Supplementary Figure 5. Purification of recombinant NADK. Protein gels indicated purification of (**left**) wild-type and (**right**) mutant (I90F) GST-NADK recombinant proteins from bacteria following induction with IPTG. E1-E5 = sequential elutions using reduced glutathione. Pellet (P) and supernatant (S) of lysed bacteria before (uniduced) and after induction (induced), small (mini), large (maxi) scale induction and flow-through lysate after GST-protein purification were loaded as indicated.

Supplementary Figure 6

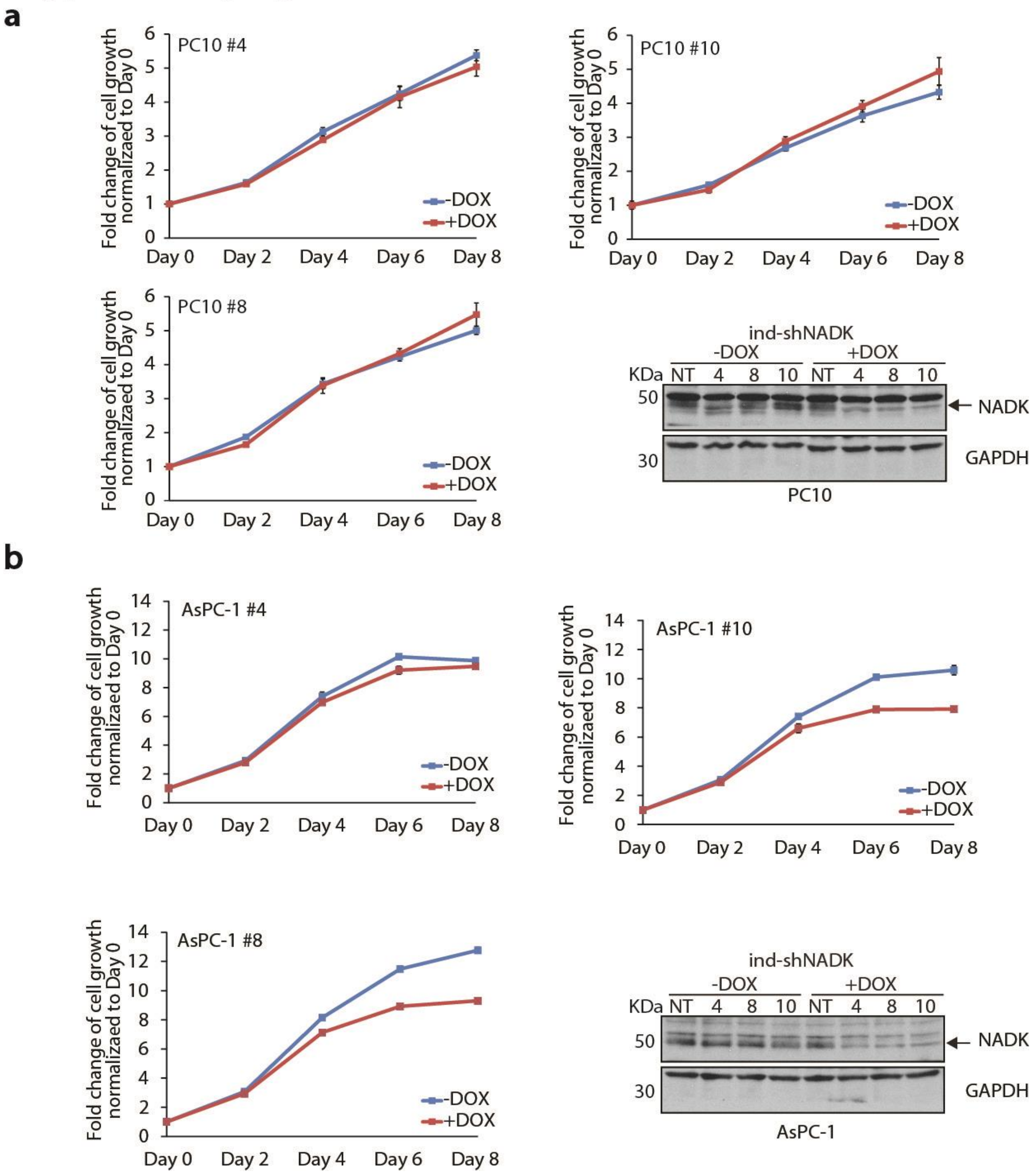

Supplementary Figure 6. NADK depletion and proliferation studies. (a) PC10 fibroblasts and (b) AsPC-1 PDAC cells were treated with Dox-inducible shRNA constructs (#4, #8, #10) were assessed for proliferation in the absence or presence of Dox. Error bars denote standard deviation (N=4 replicates per sample). Immunoblots confirming Dox-induced NADK depletion shown at right.

Supplementary Figure 7

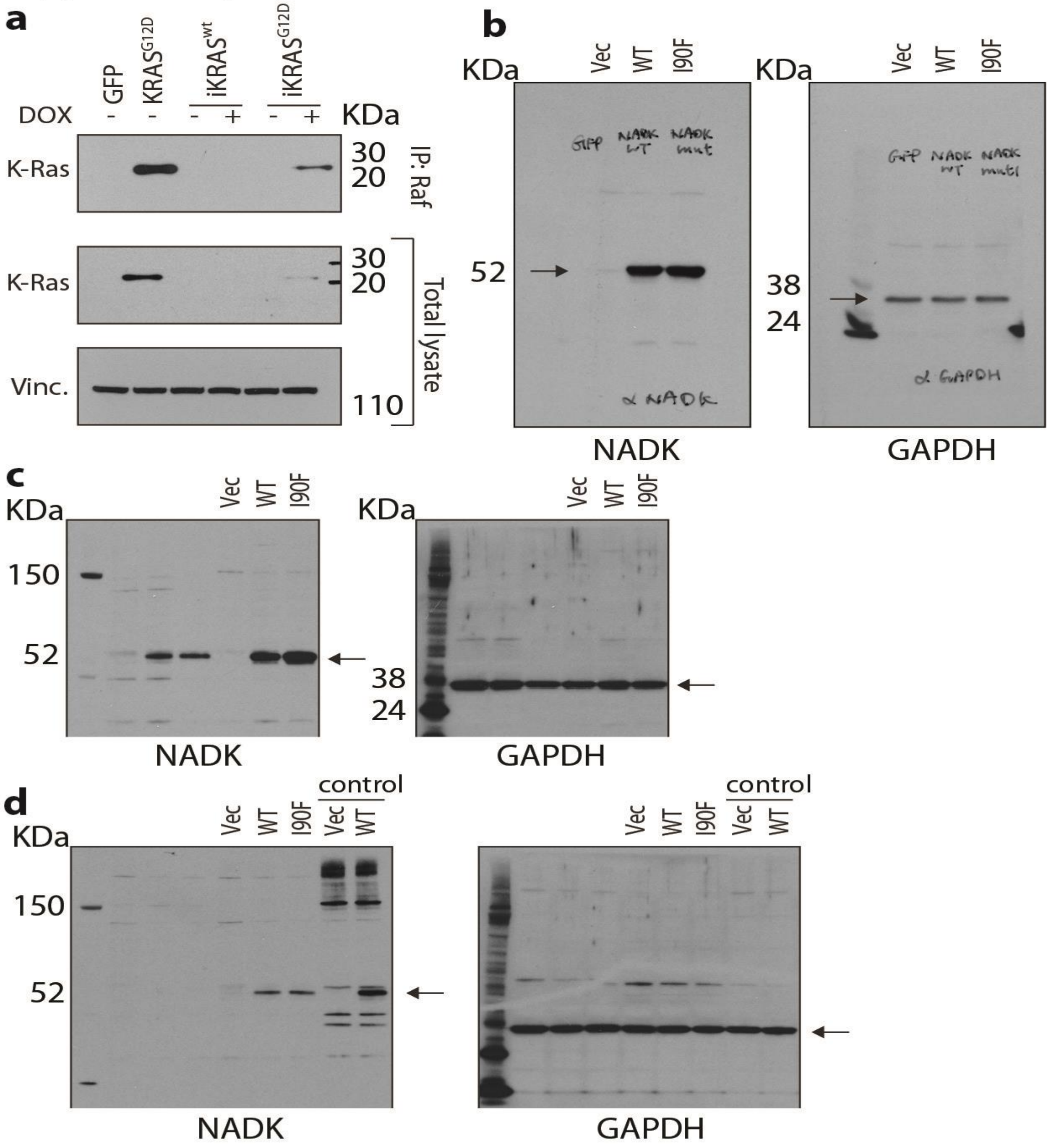

Supplementary Figure 7. Full immunoblot scans corresponding to those shown in Figures 2 and 4. Shown immunoblots correspond to (a) HPDE cells, Fig. 2b, (b) HPDE cells, Fig. 4e, (c) BxPC-3 cells, Fig. 4e, (d) Panc-1, Fig. 4e.

Supplementary Figure 8

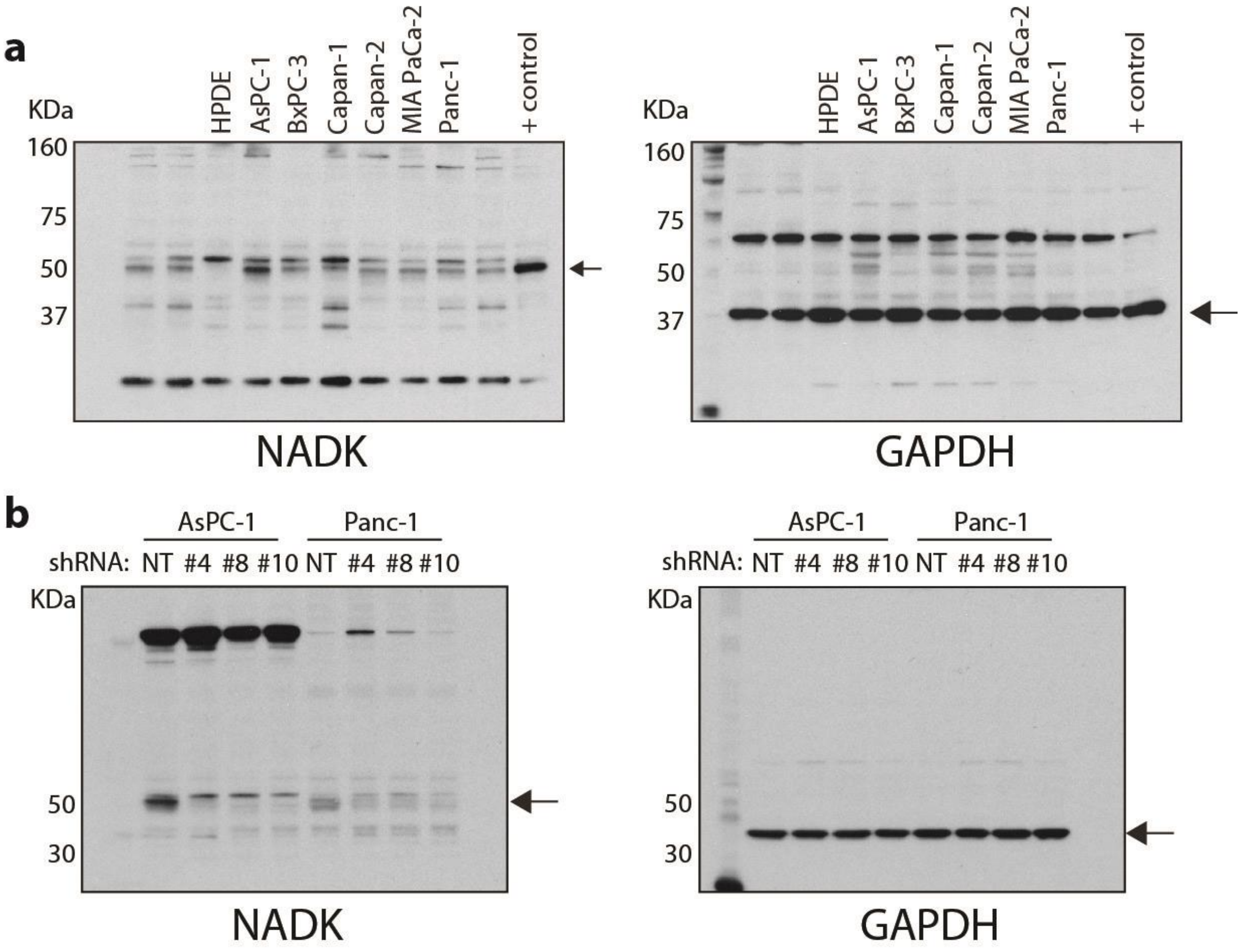

Supplementary Figure 8. Full immunoblot scans corresponding to those shown in Figure 5. Shown immunoblots correspond to (a) PDAC cell lines, Fig. 5a, (b) AsPC-1 and Panc-1 cells depleted for NADK, Fig. 5b.

Supplementary Table 1: PDAC screening aberration library.

Gene Symbol	Gene ID	Patient ID	ORF Accession	Mutation	Mutant Primer Sequence
**Computationally-Selected Aberration Set**					
ACVR1B	91	ICGC_0035_TD	NM_004302	D402E	actcctttaaatgtgctgaAatttatgccctcgggcttgtatattgggag
ACVR2A	92	ICGC_0056_TD	NM_001616	C105fs*1	ctgaagtatatttttgttgc-tgagggcaatatgtgtaatgaaaagttttc
ACVR2A	92	ICGC_0051_TD	NM_001616	C16*	ccgtctttcttatctcctgAtcttcaggtgctatacttggtagatcagaa
ARID2	196528	ICGC_0049_TD	NM_152641	R314S	agctaatcgtacctgtcttAgtttcctattactttctgcacatagtcatt
ARID2	196528	ICGC_0035_TD	NM_152641	Y939fs*12	agctcaacaaggtcaaactAtatgcaccagccattcaccaaattgttctt
BLID	414899	ICGC_0016_TD	BC130361.1	S104T	tacattgttatgcaattccActgctgaggccctgCCCAACTTTCTTGTAC
BST1	683	PCSI0044_T	NM_004334	P102T	cttgtccaggcactctattAccagagataagtccctgttctgggaaaata
BST1	683	PACA-98-T	NM_004334	A285T	tgactgtgccttaaagtcgAcagcagccgctactcaaagaaaagccccaa
CDC27	996	PCSI0044_T	NM_001114091	P316fs*27	tgtgccatccaccggagccc-ttcaaaaaagacttttcgtgttttacagtc
CDC27	996	PCSI0018_T	NM_001114091	I493V	ctgcaaagaagctataaatGttttgagccatctaccttctcaccactaca
CECR5	27440	PCSI0048_T	NM_033070	L212F	gcagctgatcatggatgtcTtcctcagcaatgggagccctggggctggcc
CECR5	27440	PCSI0018_T	NM_033070	G321V	cctatgtctgacgtatacgTcgccaacctgttccaccagtacctgcagaa
ELMOD1	55531	ICGC_0020_TD	NM_018712	V110I	ggcttgccttctgcaaatcAttgggtacaggaaccttattgcagatgtgg
ELMOD1	55531	PCSI0018_T	NM_018712	R307C	tcgtgtgagggagaaattcTgcaagaggatcatcaaacagctgcagaacc
EPC1	80314	ICGC_0056_TD	NM_025209	R182G	tgaatattggattaaaaagGgaaaaaactgtcgagggccatctcttattc
EPC1	80314	ICGC_0027_TD	NM_025209	W425G	ccaaactggcaactggcctGggactagtcctaaagatggaggattagggg
FSHR	2492	PCSI0018_T	NM_020994	L200V	ccaactagatgagctgaatGtaagcgataataataatttagaagaattgc
FSHR	2492	PACA-46-T	NM_020994	W436C	acaactatgccattgactgTcaaactggggcaggctgtgatgctgctggc
GALK2	2585	PACA-37-T	NM_002044	A164T	gacgctcacagtgctgggaTggaatctatccaaggtggaacttgcagaaa
GALK2	2585	ICGC_0059_TD	NM_002044	G403R	gtcacgacttactggagcaAgatggggaggctgcacagtatcaatggtac
HCST	10870	ICGC_0054_TD	NM_014266	T20K	ccagtggctgcagctcagaAgactccaggagagagatcatcactccctgc
ITIH5L	347365	PCSI0048_T	NM_198510	S376C	tgcttcagtgctgaaccatTgcaaccaggagcctgggaggggccccagtg
ITIH5L	347365	PCSI0048_T	NM_198510	H375Q	ctgcttcagtgctgaaccaGagcaaccaggagcctgggaggggccccagt
ITIH5L	347365	PCSI0048_T	NM_198510	H375N	agctgcttcagtgctgaacAatagcaaccaggagcctgggaggggcccca
JOSD1	9929	PCSI0072_T	NM_014876	E166*	gcccgagtggattggaggcTagagcgagctcaggaagtttctaaaacatc
MAGEA6	4105	PACA-86-T	NM_005363	N254I	caatatttcgtgcaggaaaTctacctggagtaccggcaggtccccggcag
MAGEA6	4105	ICGC_0050_TD	NM_005363	H305fs*>7	cgcatttcctacccactcct-ctttgagagagggggaagagtgaCCCAACT
MAP2K4	6416	ICGC_0032_TD	NM_003010	V151L	acttcttatggatttggatTtagtaatgcggagtagtgattgcccataca
MAP2K4	6416	PCSI0024_T	NM_003010	G303V	ttgtatgagttggccacagTccgatttccttatccaaagtggaatagtgt
MAP2K4	6416	ICGC_0037_TD	NM_003010	R281*	aatagacccaagcgcatcaTgacaaggatatgatgtccgctctgatgtct
MMP26	56547	ICGC_0030_TD	NM_021801	R63P	ctcctgcaacaattccatcCgaatgggacagacctacttgacatgcagat
MYADM	91663	PCSI0044_T	NM_001020818	A189T	cgttgcctgcatcatcttcAcgttcatcagcgaccccaacctgtaccagc
NALCN	259232	PCSI0073_T	NM_052867	M90I	cagagatgatagcaaaaatAcacatccggggcattgtcaagggggatagt
NALCN	259232	PCSI0073_T	NM_052867	H91Y	agagatgatagcaaaaatgTacatccggggcattgtcaagggggatagtt
NALCN	259232	ICGC_0052_TD	NM_052867	K498N	tagaagactttgtgtacaaTatatttggtcctggaaaaaagcttgggagt
NTF3	4908	ICGC_0046_TD	NM_001102654	R151W	cagaacatcacggcggaaaTggtacgcggagcataagagtcaccgagggg
NTF3	4908	ICGC_0013_TD	NM_001102654	R251W	caataaactcgtgggctggTggtggatacggatagacacgtcctgtgtgt
NTRK2	4915	ICGC_0029_TD	NM_001007097	W158*	gctcctgtgacattatgtgAatcaagactctccaagaggctaaatccagt
OR10K1	391109	ICGC_0015_TD	NM_001004473	R220H	atcctagtctcctacatccAcatcatctctgccattctaaaaatcccttc
OR10K1	391109	PCSI0048_T	NM_001004473	I126M	atgatcgctatatggccatGtgtaacccactgcgctactcagtgctcatg

OR4D5	219875	ICGC_0056_TD	NM_001001965	A33D	ttcttcactgttttctctgAtgtgtattttatgactgtagtgggaaacct
OR4D5	219875	ICGC_0056_TD	NM_001001965	A33T	tttcttcactgttttctctActgtgtattttatgactgtagtgggaaacc
PBLD	64081	PACA-44-T	NM_022129	A87V	catgccaccctggcttctgTagctgtgctgtttcacaaaataaaaaacat
PGA4	643847	ICGC_0064_TD	NM_014224	I266T	accatgaacggagagaccaCcgcctgtgctgagggctgccaggccattgt
PLUNC	51297	ICGC_0018_TD	NM_016583	A154V	gcaagtctgttgaggctggTtgtgaagctggacatcactgcagaaatctt
PXDN	7837	ICGC_0016_TD	NM_012293	S483S	ggcggcacctggtcctgtcTtcgggaacacttagaatctctggtgttgcc
PXDN	7837	PACA-116-T	NM_012293	G378V	cggatctcctggacgagagTtgaccgcacacccttgccagttgacccgcg
PXDN	7837	ICGC_0016_TD	NM_012293	R674Q	tacacagttgaacaggcacAggcgggagaaatctttgaacggacattgca
PXDN	7837	PACA-1130-T	NM_012293	V359I	ggtgctggttggggagagcAtcacgctggagtgcagcgccacaggccacc
RBMX	27316	PACA-46-T	NM_002139	D312N	tggaagcagtcgctatgatAattacagcagctcacgtgacggatatggtg
RBMX	27316	PCSI0019_T	NM_002139	P106fs*32	ccaagaagtagaggccctcTTcaagaggtcttagaggtggaagaggaggaa
RIPK4	54101	PCSI0073_T	NM_020639	G86V	tacatcctgcctgtgtatgTcatctgccgcgaacctgtcggcctggtcat
RIPK4	54101	PCSI0048_T	NM_020639	Q277E	gcgagttaggcccaccttcGaagaaattacttctgaaaccgaggacctgt
RSBN1L	222194	ICGC_0051_TD	NM_198467	A773V	attcctgaaaagactacagTactgaataatatggatggcaagaatgttaa
RSBN1L	222194	PACA-86-T	NM_198467	A292V	gatgttgaagatcaagcagTcaaaggcatcctaaatgataacataaaaga
SLC16A4	9122	ICGC_0044_TD	NM_004696	W61fs*22	cacctcagagcaaattggtAtggattggatccatcatgtcatctcttcgt
SMAP2	64744	ICGC_0062_TD	NM_022733	Q77H	accagtggactcaagaacaTattcagtgcatgcaagagatgggaaatgga
SMAP2	64744	PCSI0046_T	NM_022733	S180P	tcggaaaagctccccgaaaCccacagcgcctgtcatggatttgttgggcc
TGFBR2	7048	ICGC_0051_TD	NM_001024847	D471V	gagtccttcaagcagaccgTtgtctactccatggctctggtgctctggga
TGFBR2	7048	ICGC_0017_TD	NM_001024847	W480S	tccatggctctggtgctctCggaaatgacatctcgctgtaatgcagtggg
TGFBR2	7048	PACA-37-T	NM_001024847	T309T	tctttccctatgaggagtaAgcctcttggaagacagagaaggacatcttc
TGFBR2	7048	ICGC_0043_TD	NM_001024847	R520*	ggacaacgtgttgagagatTgagggcgaccagaaattcccagcttctggc
TMSL3	7117	PCSI0010_T	NM_183049	I35T	ctgccttccaaagaaacgaCtgaacaggagaagcaagcaggcgaatcgta
TMTC2	160335	PACA-46-T	NM_152588	D380E	aaaatggcattaaaaacgaAgtatcacagagaacccagcttccttctacg
TRIM63	84676	PCSI0019_T	NM_032588	E200K	ttcccgtcgagtgaccaagAagaacagtcaccaggtaaaggaagagctga
TTC14	151613	PACA-50-T	NM_133462	H690Y	gaaatacaaaaaatacgctTactctggatcacgtgatttcagtagacatg
TTC14	151613	ICGC_0046_TD	NM_133462	D695N	cgctcactctggatcacgtAatttcagtagacatgagcaaagataccgtt
WDR69	164781	ICGC_0019_TD	NM_178821	N400S	gctttcaactataaaggcaGcatagtcattacaggcagcaaggataatac
WDR69	164781	ICGC_0061_TD	NM_178821	L341V	aagaaaatgcattgccaaaGtggaaggtcatgaaggtgaaatttcaaaga
ZIM2	23619	ICGC_0061_TD	NM_015363	P115L	ctcgctgaggacaggaaacTtcacaacacaatccaggacaacatggaaaa
ZNF658	26149	ICGC_0006_TD	NR_003528	Q345fs*3	atatagtacatcagaaaaca-agctggagataaatttggtgaacataatga
ZNRF2	223082	PCSI0073_T	NM_147128	E189K	gccacgaataacctataatAaggatgtactgagtaaagatgctggggaat
**Patient-Selected Aberration Set**					
CNTN4	152330	ICGC_0050_TD	NM_175613	T488fs*50	ttgccagaagtctttctgcc-ttgaagttttctgggcctccccactggaga
CNTN4	152330	ICGC_0050_TD	NM_175612	T72fs*50	ttgccagaagtctttctgcc-ttgaagttttctgggcctccccactggaga
MAGEA6	4105	ICGC_0050_TD	NM_005363	H305fs*>7	cgcatttcctacccactcct-ctttgagagagggggaagagtgaAACCCAG
RIMBP3	85376	ICGC_0050_TD	BC035246.1	G322fs*29	gtcgccgatgccactgctgTggagcaccctattggaattctcccagctac
RPS13P8	6207	ICGC_0050_TD	NM_001017	L117fs*14	tgctaaattccgtctgattCctaatagagagccggattcaccgtttggct
ZNF556	80032	ICGC_0050_TD	NM_024967	A334T	ctcatccttacacaaacacAcgagaacgcatgctaaaaagaaacctgtga
CAPN13	92291	PACA-111-T	NM_144575	D236V	actccaagtgggccaacagTtacagcacaggcgatggagaatgggctggt
DDO	8528	PACA-111-T	NM_004032	G77R	caccagtgatgtggcagccAgaatgcttattcctcacacttatccagata
IGFBP5	3488	PACA-111-T	NM_000599	R208H	gagctcaaagccagcccacAcatggtgccccgtgctgtgtacctgcccaa
RNF213	57674	PACA-111-T	NM_020954	A531V	gggaagcagattgccgctgTgctcatgctggacagcaccttcagcatcct
SYT8	90019	PACA-111-T	NM_138567	R172Q	catgagacaaaagtgcaccAaggcacgctctgccccgtgtttgacgagac

AMBRA1	55626	PCSI0023_T	NM_017749	R662H	atgcgctaccaacagaaccAtctccgttcttccacctcctcctcttcctc
ARHGAP18	93663	PCSI0023_T	NM_033515	D640V	ggggaacgctgccttgatgTtgacacttacatgaaggatttatatcagct
CLCA1	1179	PCSI0023_T	NM_001285.3	S581F	ttgaccctgactgtcacgtTccgtgcgtccaatgctaccctgcctccaat
GRM4	2914	PCSI0023_T	NM_001038493	R483C	catctaccaataccagctgTgcaacgattctgccgagtacaaggtcattg
MAGEB10	139422	PCSI0023_T	NM_182506	P244T	gcacttcatgtttggggagAccaggaagctccttaccaaagatttggtga
MCF2L2	23101	PCSI0023_T	NM_015078	F220fs*35	gcccagatgctgcagacgtt-gggtcctgcctggccacagcagagctgccc
MTBP	27085	PCSI0023_T	NM_022045	S579*	caaaaaatgagaactggttAattacctcattcatctgaacagttgctggg
NADK	65220	PCSI0023_T	NM_023018	I90F	cccccagaccatcatgcacTttcaggaccccgcgagccagcggctgacgt
KLK9	284366	PCSI0001_T	NM_012315	P84L	ctctggaaatgggagggtcTggagcagctgttccgggttacggacttctt
NOD1	10392	PCSI0001_T	NM_006092	D294N	cgagctgcactcggacttgAacctgagccgtgtgcctgacagctcctgcc
PAPPA2	60676	PCSI0001_T	NM_021936	R205W	tcgccaagtgtggaagaggTgggcggaagatgggcagggagactccggta
PTCH2	8643	PCSI0001_T	NM_003738	G171A	gttccccttattgaaaatgCaatgattgagcggatgattgagaagctgtt
THAP10	56906	PCSI0001_T	NM_020147	Y247F	gaacagagtgatttgtcttTtatggctgtacaggtgaaagaagaaacatg

Red=failed to construct

Supplementary Table 2. Barcode enrichment summary.

Top 100 computation screen BC enrichment Summary (N=7 tumors)

Tumor	DOX	Enriched ORF	Relative abundance (%) ± Standard deviation			
			Input	Core 1	Core 2	Core 3
CT1	OFF	BST1^{P102T}	3% ± 0.3%	100% ± 0.0%	100% ± 0.1%	100% ± 0.0%
CT2	OFF	BST1^{P102T}	3% ± 0.3%	2% ± 0.0%	22% ± 1.6%	56% ± 0.6%
		BST1^{A285T}	4% ± 0.3%	98% ± 0.1%	78% ± 1.4%	39% ± 0.3%
CT3	ON>OFF	BST1^{P102T}	3% ± 0.3%	100% ± 0.0%	100% ± 0.2%	100% ± 0.1%
CT4	OFF	BST1^{P102T}	3% ± 0.3%	0% ± 0.0%	35% ± 0.1%	17% ± 0.6%
		BST1^{A285T}	4% ± 0.3%	99% ± 0.1%	64% ± 0.0%	82% ± 0.5%
CT5	OFF	BST1^{P102T}	3% ± 0.3%	3% ± 3.3%	45% ± 2.1%	2% ± 1.9%
		BST1^{A285T}	4% ± 0.3%	42% ± 50.0%	26% ± 11.9%	68% ± 33.6%
		ELMOD1^{R307C}	0% ± 0.1%	41% ± 36.1%	22% ± 10.9%	24% ± 26.1%
CT6	OFF	BST1^{P102T}	3% ± 0.3%	9% ± 7.1%	25% ± 2.3%	0% ± 0.2%
		BST1^{A285T}	4% ± 0.3%	22% ± 17.3%	9% ± 0.7%	88% ± 8.1%
		ELMOD1^{R307C}	0% ± 0.1%	43% ± 11.1%	42% ± 5.7%	10% ± 6.5%
		WDR69^{L341V}	2% ± 0.2%	10% ± 1.4%	6% ± 0.2%	1% ± 0.4%
CT7	OFF	BST1^{A285T}	4% ± 0.3%	4% ± 0.6%	86% ± 1.4%	4% ± 0.2%
		WDR69^{L341V}	2% ± 0.2%	96% ± 0.6%	11% ± 0.6%	96% ± 0.1%

Personalized screen BC enrichment Summary (N=4 tumors)

Tumor	DOX	Enriched ORF	Relative abundance (%) ± Standard deviation			
			Input	Core 1	Core 2	Core 3
PT1	OFF	MAGEA6$^{C\Delta 304}$	5% ± 0.2%	100% ± 0.1%	99% ± 0.5%	99± ± 0.4%
PT2	ON>OFF	NADK^{I90F}	14% ± 0.3%	87% ± 1.2%	99% ± 0.3%	98% ± 0.2%
		THAP10^{Y247F}	3% ± 0.1%	10% ± 0.7%	0% ± 0.0%	0% ± 0.0%
PT3	OFF	NADK^{I90F}	14% ± 0.3%	97% ± 0.4%	97% ± 0.2%	99% ± 0.2%
PT4	OFF	IGFBP5^{R208H}	3% ± 0.5%	10% ± 1.8%	30% ± 4.4%	0% ± 0.2%
		NADK^{I90F}	14% ± 0.3%	59% ± 2.1%	56% ± 3.8%	99% ± 0.8%
